# Supplementary material for: Triggers of defensive medical behaviours: a cross-sectional study among physicians in the Netherlands
Source: BMJ Open. 2019 Jun 25;9(6):e025108. doi: 10.1136/bmjopen-2018-025108 (PMC6597621; doi:10.1136/bmjopen-2018-025108)
Supplement: Supplementary data [file bmjopen-2018-025108supp001.pdf]

Appendix 1: Full survey instrument

	Strongly disagree	Disagree	Neutral	Agree	Strongly agree
Thoughts regarding potential individual consequences of malpractice litigation					
The potential consequences of justified accusations…					
1…are harmful for the reputation of a physician.	1	2	3	4	5
2…are bad for the self-confidence of a physician	1	2	3	4	5
3…are justified.	1	2	3	4	5
4…mean that physicians are being charged more often.	1	2	3	4	5
5…are a way to improve the quality of care.	1	2	3	4	5
The potential consequences of unjustified accusations…					
6…are harmful for the reputation of a physician.	1	2	3	4	5
7…are bad for the self-confidence of a physician	1	2	3	4	5
8…mean that physicians are being charged more often.	1	2	3	4	5
9…are an instrument to improve the quality of care.	1	2	3	4	5
Emotions from potential individual consequences of malpractice litigation					
The potential consequences of justified accusations…					
10…evoke in me the fear of being accused.	1	2	3	4	5
11…worry me.	1	2	3	4	5
12…make me feel angry.	1	2	3	4	5
13…make me feel stressed.	1	2	3	4	5
14…evoke a feeling of shame.	1	2	3	4	5
15…evoke a feeling of guilt.	1	2	3	4	5
16…I regard as a personal attack.	1	2	3	4	5
17…I regard as a form of justice.	1	2	3	4	5
The potential consequences of unjustified accusations…					
18…evoke in me the fear of being accused.	1	2	3	4	5
19…worry me.	1	2	3	4	5
20…make me feel angry.	1	2	3	4	5
21…make me feel stressed.	1	2	3	4	5
22…I regard as a personal attack.	1	2	3	4	5

	Never	Some-times	Fairly many times	Often	Always
Defensive medicine					
How often do you…					
23...order tests that are not clinically indicated in order to prevent a potential accusation?	1	2	3	4	5
24...carry out procedures that are probably unnecessary in order to prevent a potential accusation?	1	2	3	4	5
25...make unnecessary referrals to physicians with other specialisms to prevent a potential accusation?	1	2	3	4	5
26...prescribe unnecessary medication to prevent a potential accusation?	1	2	3	4	5
27...refuse to treat patients with complex medical problems in order to avoid a potential accusation in the event of a complication?	1	2	3	4	5
28...refuse to treat patients from which you expect an accusation in order to prevent such an accusation in the event of a complication?	1	2	3	4	5
29...avoid high-risk procedures in order to prevent a potential accusation in the event of a complication?	1	2	3	4	5
Perceived patient pressure to examine or execute treatment					
When a patient…					
30...describes their symptoms extensively in emotional words, I feel pressured to further examine or treat the patient even if it is clinically not strictly necessary.	1	2	3	4	5
31...directly or indirectly criticizes previous treatments, I feel pressured to further examine or treat the patient even if it is clinically not strictly necessary.	1	2	3	4	5
32...quotes decisions of other physicians to stress the need for intervention, I feel pressured to further examine or treat the patient even if it is clinically not strictly necessary.	1	2	3	4	5
33...requests further examination or treatment, I feel pressured to further examine or treat the patient even if it is clinically not strictly necessary.	1	2	3	4	5
Perceived patient pressure to refer the patient					
When a patient…					
34...describes their symptoms in extensive emotional words, I feel pressured to refer the patient even if it is clinically not strictly necessary.	1	2	3	4	5
35...directly or indirectly criticizes previous treatments, I feel pressured to refer the patient even if it is clinically not strictly necessary.	1	2	3	4	5
36...quotes decisions of other physicians to stress the need for intervention, I feel pressured to refer the patient even if it is clinically not strictly necessary.	1	2	3	4	5

37...requests further examination or treatment, I feel pressured to refer the patient even if it is clinically not strictly necessary.	1	2	3	4	5
Perceived patient pressure to prescribe medicine					
When a patient…					
38...describes their symptoms in extensive emotional words, I feel pressured to prescribe medicine even if it is clinically not strictly necessary.	1	2	3	4	5
39...directly or indirectly criticizes previous treatments, I feel pressured to prescribe medicine even if it is clinically not strictly necessary.	1	2	3	4	5
40...quotes decisions of other physicians to stress the need for intervention, I feel pressured to prescribe medicine even if it is clinically not strictly necessary.	1	2	3	4	5
41...requests further examination or treatment, I feel pressured to prescribe medicine even if it is clinically not strictly necessary.	1	2	3	4	5
