# Supplementary material for: Triggers of defensive medical behaviours: a cross-sectional study among physicians in the Netherlands
Source: BMJ Open. 2019 Jun 25;9(6):e025108. doi: 10.1136/bmjopen-2018-025108 (PMC6597621; doi:10.1136/bmjopen-2018-025108)
Supplement: Author's manuscript [file bmjopen-2018-025108.draft_revisions.pdf]

BMJ Open is committed to open peer review. As part of this commitment we make the peer review history of every article we publish publicly available.

When an article is published we post the peer reviewers' comments and the authors' responses online. We also post the versions of the paper that were used during peer review. These are the versions that the peer review comments apply to.

The versions of the paper that follow are the versions that were submitted during the peer review process. They are not the versions of record or the final published versions. They should not be cited or distributed as the published version of this manuscript.

BMJ Open is an open access journal and the full, final, typeset and author-corrected version of record of the manuscript is available on our site with no access controls, subscription charges or pay-per-view fees (http://bmjopen.bmj.com).

If you have any questions on BMJ Open's open peer review process please email info.bmjopen@bmj.com

BMJ Open

Why is defensive behavior so deeply rooted in medicine? A cross-sectional study among physicians and residents in the Netherlands.

Journal:	*BMJ Open*
Manuscript ID	bmjopen-2018-025108
Article Type:	Research
Date Submitted by the Author:	02-Jul-2018

Complete List of Authors:	Renkema, Erik; University of Groningen, Faculty of Economics and Business, Operations, Groningen,<br>Ahaus, Kees; University of Groningen, Faculty of Economics and Business, Operations; University of Groningen, Faculty of Economics and Business,<br>Broekhuis, Manda; University Groningen, Faculty of Economics and Business, Operations<br>Tims, Maria; VU University Amsterdam, Faculty of Economics and Business Administration, Management & Organization

Keywords:	Quality in health care < HEALTH SERVICES ADMINISTRATION & MANAGEMENT, Risk management < HEALTH SERVICES ADMINISTRATION & MANAGEMENT, MEDICAL ETHICS

SCHOLARONE™
Manuscripts

For peer review only

Why is defensive behavior so deeply rooted in medicine? A cross-sectional study among physicians and residents in the Netherlands.

Authors: Erik Renkema, MSc, PhD, Kees Ahaus, MSc, PhD, Manda Broekhuis, MSc, PhD, Maria Tims, MSc, PhD.

E. Renkema is researcher, University of Groningen, Faculty of Economics and Business, Operations Department, Groningen, the Netherlands;

K. Ahaus is professor of healthcare management, University of Groningen, Faculty of Economics and Business, Operations Department, Groningen, the Netherlands.

M. Broekhuis is professor of service operations, University of Groningen, Faculty of Economics and Business, Operations Department, Groningen, the Netherlands.

M. Tims is associate professor, VU University Amsterdam, Faculty of Economics and Business Administration, Management & Organization Department, Amsterdam, the Netherlands;

Corresponding should be addressed to Erik Renkema, University of Groningen, Faculty of Economics and Business, Operations Department, P.O. Box 800, 9700 AV, Groningen, the Netherlands; telephone: +31-6-18864251; e-mail: e.h.renkema@rug.nl.

Keywords: litigation attitude, perceived patient pressure, defensive medicine

Word count: 2992

For peer review only

Abstract

Objectives The authors investigated whether the attitudes of physicians and residents towards justified and unjustified litigation, and their perception of patient pressure in demanding care, influence their use of defensive medical behaviors.

Design Cross-sectional survey using exploratory factor analysis to determine litigation attitude and perceived patient pressure factors. Regression analyses were used to regress these factors on to defensive assurance and avoidance behaviors.

Setting Data were collected from eight Dutch hospitals.

Participants Respondents were 160 physicians and 54 residents (response rate 25%) of the hospital departments of (1) anesthesiology, (2) colon, stomach and liver diseases, (3) gynecology, (4) internal medicine, (5) neurology, and (6) surgery.

Primary outcome measures Attitudes towards justified and unjustified litigation and perceived patient pressures were measured in relation to respondents' application of defensive assurance and avoidance behaviors.

Results 'Disapproval of justified litigation' and 'Concerns about unjustified litigation' were positively related to both assurance (β=.21, $p<.01$, and β=.28, $p<.001$ respectively) and avoidance (β=.16, $p<.05$, and β=.18, $p<.05$ respectively) behaviors. 'Self-blame for justified litigation' was not significantly related to both defensive behaviors. Perceived patient pressures to refer (β=.18, $p<.05$) and to prescribe medicine (β=.23, $p<.01$) had direct positive relationships with assurance behavior, whereas perceived patient pressure to prescribe medicine was also positively related to avoidance behavior (β=.14, $p<.05$). No difference was found between physicians and residents in their defensive medical behavior.

Conclusions Physicians and residents adopted more defensive medical behaviors if they had a negative attitude towards litigation. Further, physicians and residents should be aware that

For peer review only - http://bmjopen.bmj.com/site/about/guidelines.xhtml

perceived patient pressure for care can lead to them adopting defensive behaviors that negatively affects the quality and safety of patient care.

Strengths and limitations of this study

- In this study we have measured a broad spectrum of thoughts and emotions regarding litigation, and distinguished between justified and unjustified types of litigation.
- To our knowledge this is the first study in which specific forms of patient pressure have been related to defensive medical behavior by physicians and residents.
- A drawback of the study is its reliance on self-reported perceptions and behavior.

For peer review only

For peer review only - http://bmjopen.bmj.com/site/about/guidelines.xhtml

Introduction

Defensive medicine is a widespread approach among physicians, and one that negatively affects healthcare costs[1] and the quality and safety of patient care.[2,3] It is defined as the ordering of extra tests or procedures (assurance behavior), or the avoidance of high-risk patients or procedures (avoidance behavior), primarily to reduce the risk of being held liable for malpractice.[4] Studies show that between 75% and 95% of physicians adopt defensive medical behavior.[5-9] The inappropriate ordering of invasive procedures or medicines not only costs society unnecessary expense, it also exposes patients to unnecessary health risks[9]; while the avoidance of high-risk patients or procedures reduces patients' access to health care.[9] Traditionally, the adoption of defensive medical behavior is explained by concerns about malpractice litigation.[6,7,9-12] However, there is still considerable uncertainty as to how litigation concerns affect defensive medical behavior. For example, physicians vary in their thoughts and emotions towards litigation. Some regard litigation as a form of justice or as contributing to the quality of care, while others have a more negative attitude that is associated with emotions such as fear, stress, and anger, and a low self-confidence.[13] Despite this, the extent to which attitude differences play a role in defensive medical behavior is unknown. Furthermore, physicians' attitudes may differ for litigation which they view as 'justified', e.g. when they made a mistake or were negligent, and litigation which they view as 'unjustified', e.g. following an adverse event where they do not feel they acted incorrectly. A large proportion of malpractice litigation cases can be seen as 'unjustified' since they are dismissed in court[14] with physicians' behavior not being regarded as negligent.[15] Our first objective is to identify whether different attitudes towards justified and unjustified litigation lead to differences in defensive medical behavior.

For peer review only - http://bmjopen.bmj.com/site/about/guidelines.xhtml

Similarly, the aspect of patient pressure, in combination with litigation attitude, has not been addressed in defensive medicine research. Patients have become increasingly demanding in their requests for care, and may even pressurize physicians to provide specific forms of care.[16] Perceived pressure from patients is associated with higher referral rates,[17] and an increased likelihood of examining patients and over-prescribing drugs.[7,16,18-20] What remains unknown is whether perceived patient pressure strengthens the relationship between litigation attitude and defensive medicine. Consequently, our second objective is to investigate whether perceived patient pressure drives physicians and residents to apply assurance or avoidance behaviors. Besides attending physicians, we have included resident physicians in our study. Previous research[7,8,21] has found that defensive medicine is also prevalent amongst residents. Furthermore, some studies have reported that physicians alter their teaching behavior[22] to encourage their residents to practice defensively[23] in response to the fear of malpractice liability. As such, it seems important to investigate whether litigation attitude and patient pressure play a role in the defensive medical behaviors of residents. Based on this discussion, we hypothesize the following:

Hypothesis 1: A negative attitude towards justified litigation is positively related to a) assurance and b) avoidance behaviors by physicians and residents.

Hypothesis 2: A negative attitude towards unjustified litigation is unrelated to a) assurance and b) avoidance behaviors by physicians and residents.

Hypothesis 3: Perceived patient pressure for care strengthens the positive relationship between a negative attitude towards justified litigation and the a) assurance and b) avoidance behaviors by physicians and residents.

For peer review only

For peer review only - http://bmjopen.bmj.com/site/about/guidelines.xhtml

Method

Design and study population

We collected data from physicians and residents working at eight Dutch hospitals. The hospitals are a mix of general and academic hospitals, geographically spread across the Netherlands. Prior to the data collection, the questionnaire was reviewed with one physician, and a pilot test carried out involving 32 physicians (data not included in the final analysis). We asked the boards of directors, the medical staff or patient safety departments for permission to distribute the questionnaire within their hospital in the departments of (1) anesthesiology, (2) colon, stomach and liver diseases, (3) gynecology, (4) internal medicine, (5) neurology, and (6) surgery. After approval from the head of the medical staff of the relevant department(s), an email was sent to the physicians and residents containing a short explanation of the research and a hyperlink to the electronic survey. In the email, and in the questionnaire, we explicitly stated that all the information provided would be used anonymously. Participation was voluntary. Given that neither patients nor patients' data were involved in the study, we did not require ethical approval for the study under the Dutch Law on Medical Research Involving Human Subjects (WMO).

The study sample consisted of 214 physicians and residents (response rate 25%), which is in line with other studies regarding safety attitudes and medical incidents[24,25] and can be regarded as acceptable given the professionals approached.[26] The characteristics of the respondents are provided in Table 1.

For peer review only - http://bmjopen.bmj.com/site/about/guidelines.xhtml

Table 1

Characteristics of surveyed physicians No. (% of 214)

Variables	Percentages
**Gender**	
Male	121 (56.5)
Female	93 (43.5)
**Age**	
< 30	22 (10.3)
30-40	68 (31.8)
41-50	64 (29.9)
51-60	50 (23.4)
60<	10 (4.7)
**Specialism**	
Anaesthesiology	55 (25.7)
Colon, stomach and liver	19 (8.9)
Gynaecology	29 (13.6)
Internal medicine	43 (20.1)
Neurology	20 (9.3)
Surgery	34 (15.9)
Other	14 (6.5)
**Grade**	
Physician	160 (74.8)
Resident	54 (25.2)
**Workplace**	
General hospital	132 (61.7)
Academic hospital	78 (36.4)
Other workplace	4 (1.9)
**Employment form**	
Employed	119 (55.6)
Self-employed	95 (44.4)
**Litigation experience**	
Yes	34 (15.9)
No	180 (84.1)
**Formal measures after litigation no. (% of 34)**	
Yes	4 (11.8)
No	30 (88.2)

Fourteen respondents were excluded from some of the analyses because they felt unable to answer some of the questions regarding defensive medicine and perceived patient pressure. This was because they regarded prescribing medicine as not a usual procedure within their specialism.

For peer review only - http://bmjopen.bmj.com/site/about/guidelines.xhtml

Patient and public involvement

Patients and public were not involved in the development, design, recruitment and randomization of this study.

Measures

Attitudes towards justified and unjustified litigation were measured with 22 statements derived from existing research that probed thoughts and emotions regarding the potential individual consequences of justified and unjustified litigation[13,27-29]. We asked participants to respond to each statement using a five-point Likert scale ranging from 1 (strongly disagree) to 5 (strongly agree).

We measured *perceived patient pressure* with 12 statements derived from earlier studies on perceived patient pressure[16,18-20] that were adapted to match the practices of physicians. These statements were divided into three groups of four items, addressing perceived patient pressure to examine, to refer, and to prescribe medicine. Respondents rated how frequently they experienced patient pressure with options ranging from 1 (never) to 5 (always).

Defensive medicine was measured with 7 statements derived from studies on defensive medical behavior.[8,9] We split one previously used statement (i.e. refusing to treat high-risk patients) into two separate statements (i.e. refusing to treat patients with complex medical problems, and refusing to treat patients from whom one expects an accusation) to add clarity. Response options again ranged from 1 (never) to 5 (always).

Data Analysis Strategy

Prior to testing our hypotheses, we conducted a factor analysis using SPSS Statistics 22 (IBM Corporation, Armonk, New York, USA) with Principal Axis Factoring[30] to examine the psychometric properties of the measures. The criterion for retaining factors was an

For peer review only - http://bmjopen.bmj.com/site/about/guidelines.xhtml

eigenvalue[31] >1. Items were considered to contribute sufficiently to a factor when their loading[32] was >.36. Consequently, items that loaded <.36, or cross-loaded >.36, were removed during the analysis along with two-item factors since factors containing less than three items are usually weak.[33] To ease interpretation of the factor structure, we applied oblique rotation using direct oblimin and a delta of zero.[34] Cronbach's α values were calculated for the identified factors as well as the mean scores.

Next, we examined the correlations between the control variables (gender, age, specialism, workplace, employment form, litigation experience, and formal measures after litigation) and the dependent variables (assurance and avoidance behaviors). This analysis showed that age (>60 years) and specialism (anesthesiology) correlated significantly with avoidance behavior and these were therefore included in the model. We included a dummy variable for 'grade' in the model to measure potential differences in defensive behavior between physicians and residents. Following this, we tested the hypotheses with two regression analyses in which litigation attitude and perceived patient pressure were regressed on to assurance and avoidance behaviors. Finally, we investigated the interaction between litigation attitude and perceived patient pressure using an SPSS macro designed by Hayes.[35]

Results

Exploratory factor analysis

We first conducted separate exploratory factor analyses on subsets of the data (i.e. litigation attitude, perceived patient pressure and defensive medical behavior) to determine the factor structure that best fitted each subset of items. Based on the separate factor analyses, we removed 14 items from the original dataset due to their low factor loadings, high cross-loadings, or for being part of a two-item factor. We then checked the overall factor structure

For peer review only - http://bmjopen.bmj.com/site/about/guidelines.xhtml
For peer review only

through an exploratory factor analysis of the remaining 27 items (0.785; χ^2 (351) = 2255.868, p<.001). One further item was removed during this analysis due to a low factor loading and two more items that made up a two-item factor. Table 2 shows the seven-factor solution that emerged and the corresponding reliability estimates.

For peer review only

For peer review only - http://bmjopen.bmj.com/site/about/guidelines.xhtml

Table 2

Exploratory factor analysis (N=200), Cronbach's α values and factor loadings

Item wording	Loadings						
	SBJL	DISJL	COUJL	PPREF	PPMED	ASB	AVB
α	.726	.663	.860	.772	.884	.803	.650
**Self-blame towards justified litigation (SBJL)**							
The potential consequences of justified accusations evoke a feeling of guilt	**.903**	-.038	.015	.023	-.018	.036	.021
The potential consequences of justified accusations evoke a feeling of shame	**.760**	-.046	-.072	.029	-.004	.035	.096
I regard the potential consequences of justified accusations as a personal attack	**.372**	.111	-.057	-.136	-.018	-.092	-.195
**Disapproval of justified litigation (DISJL)**							
I regard the potential consequences of a justified accusation as a form of justice (reversed)	-.098	**.643**	-.016	-.126	.018	.094	.015
The potential consequences of justified accusations are justified (reversed)	-.064	**.612**	.018	.024	-.138	-.053	.019
The potential consequences of justified accusations are a way to improve the quality of care (reversed)	.054	**.561**	.098	.089	.131	.158	-.080
The potential consequences of justified accusations make me feel angry	.111	**.485**	-.117	-.045	.055	-.039	-.005
**Concerns about unjustified litigation (COUJL)**							
The potential consequences of unjustified accusations evoke in me the fear of being accused	-.022	-.028	**-.855**	.043	-.023	.057	.070
The potential consequences of unjustified accusations worry me	.028	-.040	**-.778**	.020	-.019	.048	-.106
The potential consequences of unjustified accusations make me feel stressed	.122	.090	**-.745**	-.070	.072	-.009	-.007
**Perceived patient pressure to refer (PPREF)**							
When a patient directly or indirectly criticizes previous treatments. I feel pressured to refer the patient even if it is clinically not strictly necessary	-.018	.029	-.037	**-.761**	.024	.036	-.044
When a patient quotes decisions of other physicians to stress the need for intervention. I feel pressured to refer the patient even if it is clinically not strictly necessary	.025	-.064	-.009	**-.743**	-.102	-.026	-.055
When a patient requests further examination or treatment. I feel pressured to refer the patient even if it is clinically not strictly necessary	.070	.126	.046	**-.546**	-.175	.069	.131

For peer review only - http://bmjopen.bmj.com/site/about/guidelines.xhtml

Table 2 (continued)

Exploratory factor analysis (N=200), Cronbach's α values and factor loadings

Item wording	Loadings						
	SBJL	DISJL	COULJ	PPREF	PPMED	ASB	AVB
α	.726	.663	.860	.772	.884	.803	.650
**Perceived patient pressure to prescribe medicine (PPMED)**							
When a patient describes their symptoms in extensive emotional words. I feel pressured to prescribe medicine even if it is clinically not strictly necessary	.022	.004	-.008	.055	**-.800**	-.035	-.089
When a patient quotes decisions of other physicians to stress the need for intervention. I feel pressured to prescribe medicine even if it is clinically not strictly necessary	-.020	.002	-.023	-.083	**-.789**	.087	.044
When a patient requests further examination or treatment. I feel pressured to prescribe medicine even if it is clinically not strictly necessary	-.013	.009	-.014	-.079	**-.785**	.027	.068
When a patient directly or indirectly criticizes previous treatments. I feel pressured to prescribe medicine even if it is clinically not strictly necessary	.040	-.036	.056	-.054	**-.764**	.092	-.060
**Assurance behaviour (ASB)**							
How often do you prescribe unnecessary medication to prevent a potential accusation?	.039	.037	.098	.108	-.095	**.768**	.028
How often do you order tests that are not clinically indicated in order to prevent a potential accusation?	-.025	-.007	-.157	-.088	-.021	**.638**	-.058
How often do you carry out procedures that are probably unnecessary in order to prevent a potential accusation?	.017	.024	-.126	.008	-.063	**.626**	-.104
How often do you make unnecessary referrals to physicians with other specialisms to prevent a potential accusation?	-.016	.079	-.081	-.241	.028	**.595**	-.037
**Avoidance behaviour (AVB)**							
How often do you refuse to treat patients with complex medical problems in order to avoid a potential accusation in the event of a complication	-.041	.108	-.093	.190	-.126	-.074	**-.659**
How often do you refuse to treat patients from whom you expect an accusation in order to prevent a potential accusation in the event of a complication	.019	-.068	.025	-.060	.081	.112	**-.604**
How often do you avoid high risk procedures in order to prevent a potential accusation in the event of a complication	-.012	.013	-.002	-.092	-.040	.043	**-.592**
**Eigen values**	**1.516**	**2.235**	**1.098**	**1.250**	**3.124**	**5.295**	**1.771**
**% of variation explained (cumulative)**	**6.315**	**15.628**	**20.205**	**25.412**	**38.428**	**60.489**	**67.870**

For peer review only

In more detail, three factors relate to a physician's or resident's attitude towards litigation: self-blame for justified litigation (3 items); disapproval of justified litigation (4 items); and concerns about unjustified litigation (3 items). Two factors emerged related to perceived patient pressure: perceived patient pressure to refer (3 items); and perceived pressure to prescribe medicine (4 items).

Defensive medical behavior emerged as two distinct factors: assurance behavior (4 items); and avoidance behavior (3 items). The correlation between these two behaviors was low (r = .30; p<.01). The means (standard deviations [SD]) for assurance and avoidance behaviors were 1.76 (.57) and 1.26 (.38) respectively (see Table 3), and the analysis revealed that 177 of 200 respondents (89%) claimed to, at least sometimes, apply one or more forms of assurance behavior, and 90 of 214 respondents (42%) some form of avoidance behavior.

For peer review only

Table 3

Descriptive statistics

Variable	Var. 1	Var. 2	Var. 3	Var. 4	Var. 5	Var. 6	Var. 7	Var. 8	Var. 9	M (SD)
1. Age (1 = over 60 years; 0 = 60 years or younger)										.05 (.21)
2. Anaesthesiologist (1 = Anaest.; 0 = Other spec.)	.072									.26 (.44)
3. Physician (1 = Physician; 0 = Resident)	.129	.071								.75 (.44)
4. Self-blame towards justified litigation (SBJL)	-.148*	-.064	-.030							3.31 (.76)
5. Disapproval of justified litigation (DISJL)	-.011	.068	-.041	.092						2.51 (.59)
6. Concerns about unjustified litigation (COUJL)	.025	.140*	.017	.439**	.139*					3.98 (.77)
7. Perceived patient pressure to refer (PPREF)	.051	-.177**	.006	.223**	.154*	.215**				1.86 (.45)
8. Perceived patient pressure to prescribe medicine (PPMED)	.016	-.060	.073	.040	.041	.038	.412**			1.58 (.48)
9. Assurance behaviour (ASB)	.072	.041	.057	.134	.286**	.332**	.350**	.315**		1.76 (.57)
10. Avoidance behaviour AVB)	.236**	.225**	.039	.104	.219**	.281**	.111	.131	.297**	1.26 (.38)

** Significant at the 0.01 level (two-tailed); *Significant at the 0.05 level (two-tailed);

The means are the averages of participants' responses on the underlying items (range: 1-5)

For peer review only

For peer review only - http://bmjopen.bmj.com/site/about/guidelines.xhtml

Means for the perceived patient pressure factors were 1.86 (.45) and 1.58 (.48) respectively, and the individual items showed that 191 of 214 respondents (89%) felt, at least sometimes, pressured by their patients to refer, and 144 of 200 respondents (72%) to prescribe medicine. Perceived pressure to examine did not emerge as a distinct factor.

While most factors had an acceptable Cronbach's α (>.70), the factors related to disapproval of justified litigation and to avoidance behavior had reliability coefficients slightly below this threshold. The descriptive statistics show no signs of multicollinearity in the factor solution and support the assumption that all factors, except for self-blame for justified litigation, are significantly related to either assurance or avoidance behaviors.

Hypothesis testing

The regression analysis (Table 4 and Table 5) shows that the regression models that included litigation attitude and perceived patient pressures (model 5) were significant both for assurance behavior (F (13,186) = 5.817, p<.001) and for avoidance behavior (F (13,186) = 3.540, p<.001).

For peer review only - http://bmjopen.bmj.com/site/about/guidelines.xhtml

Table 4

Results of regression analysis for assurance behavior

Predictor	β (95% CI)					
	Model 1 (R^2=.01)	Model 2 (R^2=.01)	Model 3** (R^2=.10)	Model 4*** (R^2=.18)	Model 5*** (R^2=.28)	Model 6*** (R^2=.29)
**Age > 60 (vs. age < 60)**	.07 (-.07 to .21)	.06 (-.08 to .21)	.09 (-.05 to .23)	.06 (-.07 to .20)	.05 (-.08 to .17)	.04 (-.09 to .17)
**Anaesthesiology (vs. other specialisms)**	.04 (-.11 to .18)	.03 (-.12 to .18)	.02 (-.12 to .16)	-.03 (-.17 to .11)	.01 (-.12 to .14)	.01 (-.13 to .14)
**Grade**						
Physician (vs. resident)		.05 (-.10 to .19)	.04 (-.10 to .17)	.03 (-.10 to .16)	.02 (-.11 to .14)	.02 (-.11 to .15)
**Attitude towards justified litigation**						
Self-blame towards justified litigation (SBJL)			.12 (-.02 to .25)	-.03 (-.17 to .12)	-.06 (-.20 to .08)	-.06 (-.21 to .09)
Disapproval of justified litigation (DISJL)			.27 (.14 to .40)***	.25 (.12 to .37)***	.21 (.09 to .33)**	.21 (.08 to .33)**
**Attitude towards unjustified litigation**						
Concerns about unjustified litigation (COUJL)				.31 (.17 to .46)***	.28 (.14 to .42)***	.28 (.13 to .43)***
**Perceived patient pressure**						
Perceived patient pressure to refer (PPREF)					.18 (.03 to .33)*	.19 (.04 to .34)*
Perceived patient pressure to prescribe medicine (PPMED)					.23 (.09 to .36)**	.22 (.08 to .36)**
**Interactions**						
SBJL x PPREF						.02 (-.14 to .18)
SBJL x PPMED						.03 (-.14 to .19)
DISJL x PPREF						.00 (-.14 to .15)
DISJL x PPMED						-.03 (-.19 to .13)
COUJL x PPREF						-.03 (-.19 to .13)
COUJL x PPMED						.01 (-.17 to .16)

*** $P < 0.001$; ** $P < 0.01$; * $P < 0.05$;

For peer review only - http://bmjopen.bmj.com/site/about/guidelines.xhtml

Table 5

Results of regression analysis for avoidance behavior

	β (95% CI)					
**Predictor**	**Model 1*** (R^2=.13)**	**Model 2*** (R^2=.13)**	**Model 3*** (R^2=.19)**	**Model 4*** (R^2=.21)**	**Model 5*** (R^2=.23)**	**Model 6*** (R^2=.24)**
**Age > 60 (vs. age < 60)**	.26 (.12 to .39)***	.25 (.12 to .39)***	.28 (.15 to .41)***	.27 (.13 to .40)***	.27 (.14 to .40)***	.26 (.13 to .40)***
**Anaesthesiology (vs. other specialisms)**	.24 (.10 to .37)**	.24 (.10 to .37)**	.23 (.10 to .37)**	.20 (.07 to .34)**	.21 (.07 to .34)**	.20 (.06 to .34)**
**Grade**						
Physician (vs. resident)		.01 (-.12 to .14)	.00 (-.13 to .13)	.00 (-.13 to .13)	-.01 (-.14 to .11)	.00 (-.14 to .12)
**Attitude towards justified litigation**						
Self-blame towards justified litigation (SBJL)			.15 (.02 to .28)*	.07 (-.08 to .21)	.07 (-.08 to .21)	.07 (-.08 to .22)
Disapproval of justified litigation (DISJL)			.17 (.04 to .30)**	.16 (.03 to .28)***	.16 (.03 to .28)*	.15 (.02 to .28)*
**Attitude towards unjustified litigation**						
Concerns about unjustified litigation (COUJL)				.18 (.04 to .33)***	.18 (.04 to .33)*	.17 (.01 to .32)*
**Perceived patient pressure**						
Perceived patient pressure to refer (PPREF)					-.04 (-.19 to .11)	-.04 (-.19 to .12)
Perceived patient pressure to prescribe medicine (PPMED)					.14 (.00 to .28)*	.12 (-.03 to .26)
**Interactions**						
SBJL x PPREF						-.04 (-.20 to .13)
SBJL x PPMED						.13 (-.04 to .30)
DISJL x PPREF						-.02 (-.17 to .14)
DISJL x PPMED						-.03 (-.19 to .14)
COUJL x PPREF						.01 (-.15 to .17)
COUJL x PPMED						-.01 (-.18 to .16)

*** $P < 0.001$; ** $P < 0.01$; * $P < 0.05$;

For peer review only

For peer review only - http://bmjopen.bmj.com/site/about/guidelines.xhtml

In line with hypothesis 1a, ‘disapproval of justified litigation’ was positively related to assurance behavior (β=.21, p<.01). However, no significant relationship was found between ‘self-blame for justified litigation’ and assurance behavior (β=-.06, p=.42). Turning to hypothesis 1b, ‘disapproval of justified litigation’ (β=.16, p<.05) was positively related to avoidance behavior, providing support for hypothesis 1b, whereas ‘self-blame for justified litigation’ was not (β=.07, p<.35). The negative attitudes towards justified litigation explained 9% of the variance in assurance behavior and 6% of the variance in avoidance behavior.

In hypothesis 2, we suggested that a negative attitude towards unjustified litigation would be unrelated to a) assurance and b) avoidance behaviors. However, results showed that concerns about unjustified litigation were positively related to both assurance (β=.28, p<.001) and avoidance behaviors (β=.18, p<.05), leading us to reject hypothesis 2 (a and b). Additionally, a negative attitude towards unjustified litigation explained 8% of variance in assurance behavior and 2% of the variance in avoidance behavior.

Finally, we tested whether perceived patient pressure interacted with negative litigation attitudes in the prediction of assurance (hypothesis 3a) and avoidance (hypothesis 3b) behaviors. None of the interaction terms was significant. However, interestingly, perceived patient pressures to refer (β=.18, p<.05) and to prescribe medicine (β=.23, p<.01) had direct positive relationships with assurance behavior, explaining an additional 11% of the variance in assurance behavior. In terms of avoidance behavior, only perceived patient pressure to prescribe medicine was positively related to avoidance behavior (β=.14, p<.05).

Discussion and conclusions

Our goal was to investigate physicians’ and residents’ attitudes towards justified and unjustified litigation in relation to defensive medical behavior, and whether perceived patient

For peer review only - http://bmjopen.bmj.com/site/about/guidelines.xhtml

pressures strengthen these relationships. Despite the low mean scores for assurance and avoidance behaviors, the vast majority of the respondents in this study did admit to engaging in assurance and avoidance behaviors (89% and 42% respectively). These findings are in line with previous studies.[5-9]

Those physicians and residents who disapproved of justified litigation, were more likely to employ assurance and avoidance behaviors. A possible explanation for their disapproval is the criminalization of errors, through which mistakes can be judged as negligence, which might lead to litigation.[36] No relationship was found between self-blame for justified litigation and physicians' defensive medical behavior. This was unexpected since previous research has found that the perception of personal failure following a complaint causes shame that induces physicians to adopt defensive medicine.[37] However, our study did reveal that concerns about unjustified litigation seem to trigger avoidance and particularly assurance behaviors. It may be that physicians and residents associate unjustified litigation with stressful long-lasting litigation procedures[38] and negative media attention,[39] even if they are eventually cleared of any blame. This suggests that physicians and residents strongly fear becoming the 'second victim'[40] of an adverse event through unjustified litigation. This finding might also suggest that they fear being held accountable for what in their eyes is an unjustified litigation case, and this induces them to adopt defensive medical behaviors. If this is indeed the case, then a valuable step would be to find a way to prevent physicians and residents having to go through unjustified litigation proceedings. This could perhaps be achieved by improving the filtering out of unjustified cases at an early stage to prevent them ending up in court.

Unlike in earlier studies,[8,9,21] we did not find significant statistical differences between physicians and residents in either their attitudes towards litigation or their defensive medical

For peer review only - http://bmjopen.bmj.com/site/about/guidelines.xhtml

behavior. This finding might be explained by a recent study[23] that reported that medical residents frequently encounter defensive medicine practices and are being taught to take malpractice liability into consideration when making clinical decisions. Our findings regarding disapproval of justified litigation and concerns about unjustified litigation indicate which thoughts and emotions may be being passed on to residents during their practical training. As such, finding ways to prevent these thoughts and emotions being instilled during academic medical training is key to reducing defensive medical behavior.

We had further expected that perceived patient pressure would be a catalyst in the relationship between litigation attitude and defensive medical behavior. However, as we did not find any interaction effects, this catalytic role was not clearly evident. A possible reason for this is that both the means and the standard deviations of perceived patient pressures were low making it harder to detect a significant interaction[41]. Nevertheless, we were able to identify direct relationships between perceived patient pressure and both assurance and avoidance behaviors. That is, physicians and residents who more often perceive pressure to refer or to prescribe medicine, more often demonstrate assurance behaviors than their colleagues who perceive these pressures less frequently. This finding may indicate that physicians and residents 'give in' to the demands of their patients in order to avoid conflicts, even if this goes against their professional judgement. Using assurance behaviors may be a way to prevent a good relationship with the patient going sour. Indeed, the preservation of a good doctor-patient relationship has been suggested elsewhere as a potential explanation for defensive medical behavior.[7,42]

Although perceived pressure to prescribe medicine was related to avoidance behavior, it did not add significantly to the explained variance. This indicates that physicians and residents rarely respond to perceived patient pressures by refusing to treat patients or avoiding

For peer review only - http://bmjopen.bmj.com/site/about/guidelines.xhtml

certain procedures, maybe because they do not see this as an appropriate response. The relationship we found between perceived patient pressures and defensive medical behavior suggests that residents might be 'trained' to recognize patient pressures and accordingly act defensively. This would imply that it is important to provide training in the patient safety curriculum on communication skills to deal with perceived patient pressures and to not let these influence one's clinical decision-making. Physicians and residents should be made aware that their perceptions of patient pressures can lead them to adopt assurance and avoidance behaviors.

A strength of this study is that we have measured a broad spectrum of thoughts and emotions regarding litigation, and distinguished between justified and unjustified types of litigation. Another strength of this study is that it is the first in which specific forms of patient pressure have been related to defensive medical behavior by physicians and residents, showing that perceptions of patient pressure are crucial in determining the adoption of defensive medical behaviors. Nevertheless, our study has several limitations. First, our data were self-reported perceptions and behaviors. Although there are no obvious concerns with the data in that variables are distinguishable and correlations between them are not excessive, other data sources could be used to verify the outcomes of this study. The second limitation is the study's cross-sectional design, which makes it impossible to determine the causality of relationships. Nevertheless, the literature [43,44] suggests that attitude is an important driver of behavior and supports the direction of the relationships we hypothesized between the theoretically linked variables. We would encourage future researchers to use longitudinal studies to further investigate the relationships we found. Third, we executed our research in a context in which there is only a modest litigation climate. Given that behavior is context-

For peer review only - http://bmjopen.bmj.com/site/about/guidelines.xhtml

specific, it is reasonable to expect the relationships found in this study for defensive medical behavior to be stronger in more litigious climates.

To summarize, our study demonstrates that physicians' and residents' 'disapproval of justified litigation' and their 'concerns about unjustified litigation' stimulate both assurance and avoidance forms of defensive medical behavior, whereas 'self-blame for justified litigation' did not. Further, perceptions of patient pressure to refer and to prescribe medicine stimulate physicians and residents to adopt both avoidance and assurance behaviors that can negatively affect the quality and safety of care. To prevent physicians implicitly instilling defensive medical behavior in residents, medical curricula should specifically address litigation attitudes and how to deal with perceived patient pressures.

Acknowledgements: The authors would like to thank all physicians and residents who participated in their study.

Contributors: All authors designed the study, analyzed the data, and drafted the manuscript. ER collected the data. All authors read and approved the final manuscript.

Competing interests: The authors declare that they have no competing interests.

Funding: This research received no specific grant from any funding agency in the public, commercial or not-for-profit sectors.

Data sharing statement: No additional data are available.

For peer review only

For peer review only - http://bmjopen.bmj.com/site/about/guidelines.xhtml

References

1 Kessler D, McClellan M. Do doctors practice defensive medicine? *Q J Econ.* 1996;111:353–90.

2 Dalton GD, Samaropoulos XF, Dalton AC. Effect of physician strategies for coping with the US medical malpractice crisis on healthcare delivery and patient access to healthcare. *Public Health.* 2008;122:1051–60.

3 DeKay LM, Asch DA. Is the use of diagnostic tests good for patients, or bad? *Med Decis Making.* 1998;18:19–28.

4 US Congress, Office of Technology Assessment. *Defensive Medicine and Medical Malpractice*. Pub. No. OTA-H-602. Washington, DC: US Government Printing Office; 1994.

5 Bishop TF, Federman AD, Keyhani S. Physicians' views on defensive medicine: a national survey. *Arch Intern Med.* 2010;170:1081–3.

6 Catino M. Blame culture and defensive medicine. *Cogn Technol Work.* 2009;11:245–53.

7 He AJ. The doctor–patient relationship, defensive medicine and overprescription in Chinese public hospitals: Evidence from a cross-sectional survey in Shenzhen city. *Soc Sci Med.* 2014;123:64–71.

8 Ortashi O, Virdee J, Hassan R, et al. The practice of defensive medicine among hospital doctors in the United Kingdom. *BMC Med Ethics.* 2013;14:42.

9 Studdert DM, Mello MM, Sage WM, et al. Defensive medicine among high-risk specialist physicians in a volatile malpractice environment. *JAMA.* 2005;293:2609–17.

10 Carrier ER, Reschovsky JD, Katz DA, et al. High physician concern about malpractice risk predicts more aggressive diagnostic testing in office-based practice. *Health Aff.* 2013;32:1383–91.

For peer review only - http://bmjopen.bmj.com/site/about/guidelines.xhtml

11 Nahed BV, Babu MA, Smith TR, et al. Malpractice liability and defensive medicine: a national survey of neurosurgeons. *PloS one.* 2012;7:e39237.

12 Nakamura N, Yamashita Y. Malpractice lawsuits and change in work in Japanese surgeons. *J Surg Res.* 2015;193:210–6.

13 Renkema E, Broekhuis MH, Ahaus K. Explaining the unexplainable–the impact of physicians' attitude towards litigation on their incident disclosure behaviour. *J Eval Clin Pract.* 2014;20:649–56.

14 Jena AB, Chandra A, Lakdawalla D, et al. Outcomes of medical malpractice litigation against US physicians. *Arch Intern Med.* 2012;172:892–4.

15 Peters Jr. PG. Twenty years of evidence on the outcomes of malpractice claims. *Clin Orthop Relat Res.* 2009;467:352–7.

16 Little P, Dorward M, Warner G, et al. Importance of patient pressure and perceived pressure and perceived medical need for investigations, referral, and prescribing in primary care: nested observational study. *BMJ.* 2004;328:444.

17 Armstrong D, Fry J, Armstrong P. Doctors' perceptions of pressure from patients for referral. *BMJ.* 1991;302:1186–8.

18 Ring A, Dowrick C, Humphris G, et al. Do patients with unexplained physical symptoms pressurise general practitioners for somatic treatment? A qualitative study. *BMJ.* 2004;328:1057.

19 Salmon P, Humphris GM, Ring A, et al. Why do primary care physicians propose medical care to patients with medically unexplained symptoms? A new method of sequence analysis to test theories of patient pressure. *Psychosom Med.* 2006;68:570–7.

20 Salmon P, Marchant-Haycox S. Surgery in the absence of pathology The relationship of patients' presentation to gynecologists' decisions for hysterectomy. *J Psychosom Res.* 2000;49:119–24.

For peer review only - http://bmjopen.bmj.com/site/about/guidelines.xhtml

21 Passmore K, Leung W-C. Defensive practice amongst psychiatrists: a questionnaire survey. *Postgrad Med J.* 2002;78:671–3.

22 Reed DA, Windish DM, Levine RB, Kravet KJ, Wolfe L, Wright SM. Do fears of malpractice litigation influence teaching behaviors? *Teach Learn Med.* 2008;20:205-11.

23 O'Leary KJ, Choi J, Watson K, Williams MV. Medical students' and residents' clinical and educational experiences with defensive medicine. *Acad Med.* 2012;87:142-8.

24 Cole AP, Block L, Wu AW. On higher ground: ethical reasoning and its relationship with error disclosure. *BMJ Qual Saf.* 2013;22:580–5.

25 Durani P, Dias J, Singh HP, Taub N. Junior doctors and patient safety: evaluating knowledge, attitudes and perception of safety climate. *BMJ Qual Saf.* 2013;22:65–71.

26 Baruch Y, Holtom BC. Survey response rate levels and trends in organizational research. *Hum Relat.* 2008;61:1139–60.

27 Bark P, Vincent C, Olivieri L, et al. Impact of litigation on senior clinicians: implications for risk management. *Qual Saf Health Care.* 1997;6:7–13.

28 Benbassat J, Pilpel D, Schor R. Physicians' attitudes toward litigation and defensive practice: development of scale. *Behav Med.* 2001;27:52–60.

29 Bovbjerg RR, Miller RH, Shapiro DW. Paths to reducing medical injury: professional liability and discipline vs. patient safety – and the need for a third way. *J Law Med Ethics.* 2001;29:369–380.

30 De Winter JC, Dodou D. Factor recovery by principal axis factoring and maximum likelihood factor analysis as a function of factor pattern and sample size. *J Appl Stat.* 2012;39:695–710.

31 Kaiser HF. The application of electronic computers to factor analysis. *Educ Psychol Meas.* 1960:20:141–151.

For peer review only - http://bmjopen.bmj.com/site/about/guidelines.xhtml

32 Stevens JP. *Applied multivariate statistics for the social sciences* (fifth edition). New York, NY: Routledge; 2009.

33 Osborne JW, Costello AB. Best practices in exploratory factor analysis: Four recommendations for getting the most from your analysis. *Pan-Pacific Management Review* 2009;12:131–146.

34 Field A. *Discovering statistics using IBM SPSS statistics.* Thousand Oaks, CA: Sage; 2013.

35 Hayes, A.F. (2018). *Introduction to mediation, moderation, and conditional process analysis: A regression-based approach* (2nd Edition). New York, NY: Guilford Press; 2018.

36 Dekker S. *Just culture: Balancing safety and accountability.* Burlington, VT: Ashgate; 2007.

37 Cunningham W, Wilson H. Complaints, shame and defensive medicine. *BMJ Qual Saf.* 2011;20:449–52.

38 Seabury SA, Chandra A, Lakdwalla DN, Jena AB. On average, physicians spend nearly 11 percent of their 40-year careers with an open, unresolved malpractice claim. *Health Aff* . 2013;32:111–9.

39 Dekker, Sidney. *Second victim: Error, guilt, trauma, and resilience.* Boca Raton, FL: CRC press; 2013.

40 Scott SD, Hirschinger LE, Cox KR, et al. The natural history of recovery for the healthcare provider “second victim” after adverse patient events. *Qual Saf Health Care.* 2009;18:325–30.

41 Stone-Romero EF, Liakhovitski D. Strategies for detecting moderator variables: A review of conceptual and empirical issues. *Res Person Hum Resour Man.* 2002;21:333–72.

For peer review only - http://bmjopen.bmj.com/site/about/guidelines.xhtml

42 Cunningham W, Dovey S. Defensive changes in medical practice and the complaints process: a qualitative study of New Zealand doctors. *New Zeal Med J.* 2006;119.

43 Ajzen I. Nature and operation of attitudes. *Annu Rev Psychol.* 2001;52:27–58.

44 Loewenstein GF, Weber EU, Hsee CK, et al. Risk as feelings. *Psychol Bull.* 2001;127:267.

For peer review only

For peer review only - http://bmjopen.bmj.com/site/about/guidelines.xhtml

Why is defensive behavior so deeply rooted in medicine? A cross-sectional study among physicians and residents in the Netherlands.

Journal:	*BMJ Open*
Manuscript ID	bmjopen-2018-025108.R1
Article Type:	Research
Date Submitted by the Author:	24-Oct-2018
Complete List of Authors:	Renkema, Erik; University of Groningen, Faculty of Economics and Business, Operations<br>Ahaus, Kees; University of Groningen, Faculty of Economics and Business, Operations<br>Broekhuis, Manda; University of Groningen, Faculty of Economics and Business, Operations<br>Tims, Maria; VU University Amsterdam, Faculty of Economics and Business Administration, Management & Organization
**Primary Subject Heading**:	Health services research
Secondary Subject Heading:	Patient-centred medicine, Ethics
Keywords:	Quality in health care < HEALTH SERVICES ADMINISTRATION & MANAGEMENT, Risk management < HEALTH SERVICES ADMINISTRATION & MANAGEMENT, MEDICAL ETHICS

For peer review only - http://bmjopen.bmj.com/site/about/guidelines.xhtml

Why is defensive behavior so deeply rooted in medicine? A cross-sectional study among physicians and residents in the Netherlands.

Authors: Erik Renkema, MSc, PhD, Kees Ahaus, MSc, PhD, Manda Broekhuis, MSc, PhD, Maria Tims, MSc, PhD.

E. Renkema is researcher, University of Groningen, Faculty of Economics and Business, Operations Department, Groningen, the Netherlands;

K. Ahaus is professor of healthcare management, University of Groningen, Faculty of Economics and Business, Operations Department, Groningen, the Netherlands.

M. Broekhuis is professor of professional service chains, University of Groningen, Faculty of Economics and Business, Operations Department, Groningen, the Netherlands.

M. Tims is associate professor, VU University Amsterdam, Faculty of Economics and Business Administration, Management & Organization Department, Amsterdam, the Netherlands;

Corresponding should be addressed to Erik Renkema, University of Groningen, Faculty of Economics and Business, Operations Department, P.O. Box 800, 9700 AV, Groningen, the Netherlands; telephone: +31-6-18864251; e-mail: e.h.renkema@rug.nl.

Keywords: litigation attitude, perceived patient pressure, defensive medicine

Word count: 3192

For peer review only

Abstract

Objectives This study investigated whether the attitudes of physicians and residents towards justified and unjustified litigation, and their perception of patient pressure in demanding care, influence their use of defensive medical behaviors.

Design Cross-sectional survey using exploratory factor analysis to determine litigation attitude and perceived patient pressure factors. Regression analyses were used to regress these factors on to defensive assurance and avoidance behaviors.

Setting Data were collected from eight Dutch hospitals.

Participants Respondents were 160 physicians and 54 residents (response rate 25%) of the hospital departments of (1) anesthesiology, (2) colon, stomach and liver diseases, (3) gynecology, (4) internal medicine, (5) neurology, and (6) surgery.

Primary outcome measures Attitudes towards justified and unjustified litigation and perceived patient pressures were measured in relation to respondents' application of defensive assurance and avoidance behaviors.

Results 'Disapproval of justified litigation' and 'Concerns about unjustified litigation' were positively related to both assurance ($\beta=.21$, $p<.01$, and $\beta=.28$, $p<.001$ respectively) and avoidance ($\beta=.16$, $p<.05$, and $\beta=.18$, $p<.05$ respectively) behaviors. 'Self-blame for justified litigation' was not significantly related to both defensive behaviors. Perceived patient pressures to refer ($\beta=.18$, $p<.05$) and to prescribe medicine ($\beta=.23$, $p<.01$) had direct positive relationships with assurance behavior, whereas perceived patient pressure to prescribe medicine was also positively related to avoidance behavior ($\beta=.14$, $p<.05$). No difference was found between physicians and residents in their defensive medical behavior.

For peer review only - http://bmjopen.bmj.com/site/about/guidelines.xhtml

Conclusions Physicians and residents adopted more defensive medical behaviors if they had a negative attitude towards litigation. Further, physicians and residents should be aware that perceived patient pressure for care can lead to them adopting defensive behaviors that negatively affects the quality and safety of patient care.

Strengths and limitations of this study

- In this study a broad spectrum of thoughts and emotions regarding litigation were measured, and a distinction was made between justified and unjustified types of litigation.
- This is the first study in which specific forms of patient pressure have been related to physicians' and residents' defensive medical behavior.
- A drawback of the study is its reliance on self-reported perceptions and behavior.

For peer review only

For peer review only - http://bmjopen.bmj.com/site/about/guidelines.xhtml

Introduction

Defensive medicine is a widespread approach among physicians, and one that negatively affects healthcare costs[1] and the quality and safety of patient care.[2,3] It is defined as the ordering of extra tests or procedures (assurance behavior), or the avoidance of high-risk patients or procedures (avoidance behavior), primarily to reduce the risk of being held liable for malpractice.[4,5] Studies show that between 60% and 95% of physicians adopt defensive medical behavior.[4,6-11] The inappropriate ordering of invasive procedures or medicines not only costs society unnecessary expense[4], it also exposes patients to unnecessary health risks[11]; while the avoidance of high-risk patients or procedures reduces patients' access to health care.[11] Traditionally, the adoption of defensive medical behavior is explained by concerns about malpractice litigation.[8,9,11-15] However, there is still considerable uncertainty as to how litigation concerns affect defensive medical behavior. For example, physicians vary in their thoughts and emotions towards litigation. Some regard litigation as a form of justice or as contributing to the quality of care, while others have a more negative attitude that is associated with emotions such as fear, stress, and anger, and a low self-confidence.[16] Despite these differences in how physicians think and feel about litigation and what they consider as justified and unjustified, the extent to which attitude differences play a role in defensive medical behavior is unknown. Furthermore, physicians' attitudes may differ for litigation which they view as 'justified', e.g. when they made a mistake or were negligent, and litigation which they view as 'unjustified', e.g. following an adverse event where they do not feel they acted incorrectly. A large proportion of malpractice litigation cases can be seen as 'unjustified' since they are dismissed in court[17] with physicians' behavior not being regarded as negligent.[18] The first objective of this study is to identify whether different

For peer review only - http://bmjopen.bmj.com/site/about/guidelines.xhtml

attitudes towards justified and unjustified litigation lead to differences in defensive medical behavior.

Similarly, the aspect of patient pressure, in combination with litigation attitude, has not been addressed in defensive medicine research. Patients have become increasingly demanding in their requests for care, and may even pressurize physicians to provide specific forms of care.[19-21] Perceived pressure from patients is associated with higher referral rates,[22,23] and an increased likelihood of examining patients and over-prescribing drugs.[9,21,24-27] What remains unknown is whether perceived patient pressure strengthens the relationship between litigation attitude and defensive medicine. Consequently, the second objective of this study is to investigate whether perceived patient pressure drives physicians and residents to apply assurance or avoidance behaviors. Besides attending physicians, resident physicians were included in the study. Previous research[9,10,28] has found that defensive medicine is also prevalent amongst residents. Furthermore, some studies have reported that physicians alter their teaching behavior[29] to encourage their residents to practice defensively[30] in response to the fear of malpractice liability. As such, it seems important to investigate whether litigation attitude and patient pressure play a role in the defensive medical behaviors of residents. Based on this discussion, the following hypotheses have been formulated:

Hypothesis 1: A negative attitude towards justified litigation is positively related to a) assurance and b) avoidance behaviors by physicians and residents.

Hypothesis 2: A negative attitude towards unjustified litigation is unrelated to a) assurance and b) avoidance behaviors by physicians and residents.

For peer review only

For peer review only - http://bmjopen.bmj.com/site/about/guidelines.xhtml

Hypothesis 3: Perceived patient pressure for care strengthens the positive relationship between a negative attitude towards justified litigation and the a) assurance and b) avoidance behaviors by physicians and residents.

Method

Study design and sample

Between September 2014 and March 2015 a cross-sectional survey was conducted to investigate the relation between physicians' and residents' attitudes towards justified and unjustified litigation, their perception of patient pressure in demanding care, and their use of defensive medical behaviors.

Data were collected from physicians and residents working at eight Dutch hospitals. The hospitals are a mix of general and academic hospitals, geographically spread across the Netherlands. Prior to the data collection, the questionnaire was reviewed with one physician, and a pilot test carried out involving 32 physicians (data not included in the final analysis). The boards of directors, the medical staff or patient safety departments were asked for permission to distribute the questionnaire within their hospital in the departments of (1) anesthesiology, (2) colon, stomach and liver diseases, (3) gynecology, (4) internal medicine, (5) neurology, and (6) surgery. These specialisms were selected as they are relatively vulnerable to patients' complaints and litigation. After approval from the head of the medical staff of the relevant department(s), an email was sent to the physicians and residents containing a short explanation of the research and a hyperlink to the electronic survey. In the email, and in the questionnaire, it was explicitly stated that all the information provided would be used anonymously. Participation was voluntary. Given

For peer review only - http://bmjopen.bmj.com/site/about/guidelines.xhtml

that neither patients nor patients' data were involved in the study, ethical approval for the study was not required under the Dutch Law on Medical Research Involving Human Subjects (WMO).

Patient and public involvement

Patients and public were not involved in the development, design, recruitment and randomization of this study.

Measures

Attitudes towards justified and unjustified litigation were measured with 22 statements derived from existing research that probed thoughts and emotions regarding the potential individual consequences of justified and unjustified litigation[16,31-33]. Participants were asked about their emotions (e.g. fear, worry, stress) and thoughts about potential consequences of accusations. Participants were asked to respond to each statement using a five-point Likert scale ranging from 1 (strongly disagree) to 5 (strongly agree). See online supplementary appendix 1 for the full survey instrument.

Perceived patient pressure was measured with 12 statements derived from earlier studies on perceived patient pressure[21,25-27] that were adapted to match the practices of physicians. These statements were divided into three groups of four items, addressing perceived patient pressure to examine, to refer, and to prescribe medicine. Respondents rated how frequently they experienced patient pressure with options ranging from 1 (never) to 5 (always).

Defensive medicine was measured with 7 statements derived from studies on defensive medical behavior.[10,11] Participants were asked for assurance behaviors, such as prescribing unnecessary medication, ordering tests that are not clinically indicated, carrying out unnecessary procedures and making unnecessary referrals. They were also asked for avoidance behaviors,

For peer review only - http://bmjopen.bmj.com/site/about/guidelines.xhtml

such as refusing to apply high-risk procedures. One previously used avoidance behavior statement (i.e. refusing to treat high-risk patients) was split into two separate statements (i.e. refusing to treat patients with complex medical problems, and refusing to treat patients from whom one expects an accusation) to add clarity. Response options again ranged from 1 (never) to 5 (always).

Data Analysis Strategy

Prior to testing the hypotheses, a factor analysis was conducted using SPSS Statistics 22 (IBM Corporation, Armonk, New York, USA) with Principal Axis Factoring[34] to examine the psychometric properties of the measures. The criterion for retaining factors was an eigenvalue[35] >1. Items were considered to contribute sufficiently to a factor when their loading[36] was >.36. Consequently, items that loaded <.36, or cross-loaded >.36, were removed during the analysis along with two-item factors since factors containing less than three items are usually weak.[37] To ease interpretation of the factor structure, we applied oblique rotation using direct oblimin and a delta of zero.[38] Cronbach's α values were calculated for the identified factors as well as the mean scores and standard deviations. Listwise deletion was applied in all analyses.

First, separate exploratory factor analyses were conducted on subsets of the data (i.e. litigation attitude, perceived patient pressure and defensive medical behavior) to determine the factor structure that best fitted each subset of items. Based on the separate factor analyses, 14 items were removed from the original dataset due to their low factor loadings, high cross-loadings, or for being part of a two-item factor. Then the overall factor structure was checked through an exploratory factor analysis of the remaining 27 items (0.785; χ^2 (351) = 2255.868, p<.001). One further item was removed during this analysis due to a low factor loading and two

For peer review only - http://bmjopen.bmj.com/site/about/guidelines.xhtml

more items that made up a two-item factor. Table 1 shows the seven-factor solution that emerged and the corresponding reliability estimates.

For peer review only - http://bmjopen.bmj.com/site/about/guidelines.xhtml

Table 1

Exploratory factor analysis (N=200), Cronbach's α values and factor loadings

Item wording	Loadings						
	SBJL	DISJL	COUJL	PPREF	PPMED	ASB	AVB
α	.726	.663	.860	.772	.884	.803	.650
**Self-blame towards justified litigation (SBJL)**							
The potential consequences of justified accusations evoke a feeling of guilt	**.903**	-.038	.015	.023	-.018	.036	.021
The potential consequences of justified accusations evoke a feeling of shame	**.760**	-.046	-.072	.029	-.004	.035	.096
I regard the potential consequences of justified accusations as a personal attack	**.372**	.111	-.057	-.136	-.018	-.092	-.195
**Disapproval of justified litigation (DISJL)**							
I regard the potential consequences of a justified accusation as a form of justice (reversed)	-.098	**.643**	-.016	-.126	.018	.094	.015
The potential consequences of justified accusations are justified (reversed)	-.064	**.612**	.018	.024	-.138	-.053	.019
The potential consequences of justified accusations are a way to improve the quality of care (reversed)	.054	**.561**	.098	.089	.131	.158	-.080
The potential consequences of justified accusations make me feel angry	.111	**.485**	-.117	-.045	.055	-.039	-.005
**Concerns about unjustified litigation (COUJL)**							
The potential consequences of unjustified accusations evoke in me the fear of being accused	-.022	-.028	**-.855**	.043	-.023	.057	.070
The potential consequences of unjustified accusations worry me	.028	-.040	**-.778**	.020	-.019	.048	-.106
The potential consequences of unjustified accusations make me feel stressed	.122	.090	**-.745**	-.070	.072	-.009	-.007
**Perceived patient pressure to refer (PPREF)**							
When a patient directly or indirectly criticizes previous treatments. I feel pressured to refer the patient even if it is clinically not strictly necessary	-.018	.029	-.037	**-.761**	.024	.036	-.044
When a patient quotes decisions of other physicians to stress the need for intervention. I feel pressured to refer the patient even if it is clinically not strictly necessary	.025	-.064	-.009	**-.743**	-.102	-.026	-.055
When a patient requests further examination or treatment. I feel pressured to refer the patient even if it is clinically not strictly necessary	.070	.126	.046	**-.546**	-.175	.069	.131

For peer review only - http://bmjopen.bmj.com/site/about/guidelines.xhtml

Table 1 (continued)

Exploratory factor analysis (N=200), Cronbach's α values and factor loadings

Item wording		Loadings						
		SBJL	DISJL	COULJ	PPREF	PPMED	ASB	AVB
	α	.726	.663	.860	.772	.884	.803	.650
**Perceived patient pressure to prescribe medicine (PPMED)**								
When a patient describes their symptoms in extensive emotional words. I feel pressured to prescribe medicine even if it is clinically not strictly necessary		.022	.004	-.008	.055	**-.800**	-.035	-.089
When a patient quotes decisions of other physicians to stress the need for intervention. I feel pressured to prescribe medicine even if it is clinically not strictly necessary		-.020	.002	-.023	-.083	**-.789**	.087	.044
When a patient requests further examination or treatment. I feel pressured to prescribe medicine even if it is clinically not strictly necessary		-.013	.009	-.014	-.079	**-.785**	.027	.068
When a patient directly or indirectly criticizes previous treatments. I feel pressured to prescribe medicine even if it is clinically not strictly necessary		.040	-.036	.056	-.054	**-.764**	.092	-.060
**Assurance behavior (ASB)**								
How often do you prescribe unnecessary medication to prevent a potential accusation?		.039	.037	.098	.108	-.095	**.768**	.028
How often do you order tests that are not clinically indicated in order to prevent a potential accusation?		-.025	-.007	-.157	-.088	-.021	**.638**	-.058
How often do you carry out procedures that are probably unnecessary in order to prevent a potential accusation?		.017	.024	-.126	.008	-.063	**.626**	-.104
How often do you make unnecessary referrals to physicians with other specialisms to prevent a potential accusation?		-.016	.079	-.081	-.241	.028	**.595**	-.037
**Avoidance behavior (AVB)**								
How often do you refuse to treat patients with complex medical problems in order to avoid a potential accusation in the event of a complication		-.041	.108	-.093	.190	-.126	-.074	**-.659**
How often do you refuse to treat patients from whom you expect an accusation in order to prevent a potential accusation in the event of a complication		.019	-.068	.025	-.060	.081	.112	**-.604**
How often do you avoid high-risk procedures in order to prevent a potential accusation in the event of a complication		-.012	.013	-.002	-.092	-.040	.043	**-.592**
**Eigen values**		**1.516**	**2.235**	**1.098**	**1.250**	**3.124**	**5.295**	**1.771**
**% of variation explained (cumulative)**		**6.315**	**15.628**	**20.205**	**25.412**	**38.428**	**60.489**	**67.870**

For peer review only - http://bmjopen.bmj.com/site/about/guidelines.xhtml

In more detail, three factors relate to a physician's or resident's attitude towards litigation: self-blame for justified litigation (3 items); disapproval of justified litigation (4 items); and concerns about unjustified litigation (3 items). Two factors emerged related to perceived patient pressure: perceived patient pressure to refer (3 items); and perceived pressure to prescribe medicine (4 items).

Defensive medical behavior emerged as two distinct factors: assurance behavior (4 items); and avoidance behavior (3 items). The correlation between these two behaviors was low (r = .30; p<.01).

Each quantitative variable was summarized as a mean value and standard deviation. Next, the correlations between the control variables (gender, age, specialism, workplace, employment form, litigation experience, and formal measures after litigation) and the dependent variables (assurance and avoidance behaviors) were examined. This analysis showed that age (>60 years) and specialism (anesthesiology) correlated significantly with avoidance behavior and these were therefore included in the model as control variables. A dummy variable was included for 'grade' in the model to measure potential differences in defensive behavior between physicians and residents. Following this, the hypotheses were tested with two multiple regression analyses in which litigation attitude and perceived patient pressure were regressed on to assurance and avoidance behaviors. Finally, the interaction between litigation attitude and perceived patient pressure was investigated using a SPSS macro designed by Hayes.[39]

Results

A total of 238 surveys were received. Due to missing data of 24 participants, the study sample consisted of 214 physicians and residents (response rate 25%), which is in line with other

For peer review only - http://bmjopen.bmj.com/site/about/guidelines.xhtml

studies regarding safety attitudes and medical incidents[40,41] and can be regarded as acceptable given the professionals approached.[42] The characteristics of the respondents are provided in Table 2.

Table 2

Characteristics of the survey participants (N=214)

Variables	No. of respondents (%)
**Gender**	
Male	121 (56.5)
Female	93 (43.5)
**Age**	
< 30	22 (10.3)
30-40	68 (31.8)
41-50	64 (29.9)
51-60	50 (23.4)
60<	10 (4.7)
**Specialism**	
Anesthesiology	55 (25.7)
Colon, stomach and liver	19 (8.9)
Gynecology	29 (13.6)
Internal medicine	43 (20.1)
Neurology	20 (9.3)
Surgery	34 (15.9)
Other	14 (6.5)
**Grade**	
Physician	160 (74.8)
Resident	54 (25.2)
**Workplace**	
General hospital	132 (61.7)
Academic hospital	78 (36.4)
Other workplace	4 (1.9)
**Employment form**	
Employed	119 (55.6)
Self-employed	95 (44.4)
**Litigation experience**	
Yes	34 (15.9)
No	180 (84.1)
**Formal measures after litigation (of N=34 with litigation experience)**	
Yes	4 (11.8)
No	30 (88.2)

For peer review only - http://bmjopen.bmj.com/site/about/guidelines.xhtml

Fourteen respondents were excluded from some of the analyses because they felt unable to answer some of the questions regarding defensive medicine and perceived patient pressure. This was because they regarded prescribing medicine as not a usual procedure within their specialism.

The means (standard deviations [SD]) of all variables and the correlations between the variables are displayed in Table 3. The analysis revealed that 177 of 200 respondents (89%) claimed to, at least sometimes, apply one or more forms of assurance behavior, and 90 of 214 respondents (42%) some form of avoidance behavior.

For peer review only

For peer review only - http://bmjopen.bmj.com/site/about/guidelines.xhtml

Table 3

Descriptive statistics

Variable	Var. 1	Var. 2	Var. 3	Var. 4	Var. 5	Var. 6	Var. 7	Var. 8	Var. 9	M (SD)
1. Age (1 = over 60 years; 0 = 60 years or younger)										.05 (.21)
2. Anesthesiologist (1 = Anest.; 0 = Other spec.)	.072									.26 (.44)
3. Physician (1 = Physician; 0 = Resident)	.129	.071								.75 (.44)
4. Self-blame towards justified litigation (SBJL)	-.148*	-.064	-.030							3.31 (.76)
5. Disapproval of justified litigation (DISJL)	-.011	.068	-.041	.092						2.51 (.59)
6. Concerns about unjustified litigation (COUJL)	.025	.140*	.017	.439**	.139*					3.98 (.77)
7. Perceived patient pressure to refer (PPREF)	.051	-.177**	.006	.223**	.154*	.215**				1.86 (.45)
8. Perceived patient pressure to prescribe medicine (PPMED)	.016	-.060	.073	.040	.041	.038	.412**			1.58 (.48)
9. Assurance behavior (ASB)	.072	.041	.057	.134	.286**	.332**	.350**	.315**		1.76 (.57)
10. Avoidance behavior AVB)	.236**	.225**	.039	.104	.219**	.281**	.111	.131	.297**	1.26 (.38)

** Significant at the 0.01 level (two-tailed); *Significant at the 0.05 level (two-tailed);

The means are the averages of participants' responses on the underlying items (range: 1-5)

For peer review only

For peer review only - http://bmjopen.bmj.com/site/about/guidelines.xhtml

Means for the perceived patient pressure factors were 1.86 (.45) and 1.58 (.48) respectively, and the individual items showed that 191 of 214 respondents (89%) felt, at least sometimes, pressured by their patients to refer, and 144 of 200 respondents (72%) to prescribe medicine. Perceived pressure to examine did not emerge as a distinct factor.

While most factors had an acceptable Cronbach's α (>.70), the factors related to disapproval of justified litigation and to avoidance behavior had reliability coefficients slightly below this threshold. The descriptive statistics show no signs of multicollinearity in the factor solution and support the assumption that all factors, except for self-blame for justified litigation, are significantly related to either assurance or avoidance behaviors. Factors identified in the exploratory factor analysis were entered into a multiple linear regression model and evaluated as possible predictors of both assurance and avoidance defensive medicine behavior.

Hypothesis testing

The regression analysis (Table 4 and Table 5) shows that the regression models that included litigation attitude and perceived patient pressures (model 5) were significant both for assurance behavior (F (13,186) = 5.817, p<.001) and for avoidance behavior (F (13,186) = 3.540, p<.001).

For peer review only

Table 4

Results of regression analysis for assurance behavior

Predictor	β (95% CI)					
	**Model 1 (R^2=.01)**	**Model 2 (R^2=.01)**	**Model 3** (R^2=.10)**	**Model 4*** (R^2=.18)**	**Model 5*** (R^2=.28)**	**Model 6*** (R^2=.29)**
**Age > 60 (vs. age < 60)**	.07 (-.07 to .21)	.06 (-.08 to .21)	.09 (-.05 to .23)	.06 (-.07 to .20)	.05 (-.08 to .17)	.04 (-.09 to .17)
**Anesthesiology (vs. other specialisms)**	.04 (-.11 to .18)	.03 (-.12 to .18)	.02 (-.12 to .16)	-.03 (-.17 to .11)	.01 (-.12 to .14)	.01 (-.13 to .14)
**Grade**						
Physician (vs. resident)		.05 (-.10 to .19)	.04 (-.10 to .17)	.03 (-.10 to .16)	.02 (-.11 to .14)	.02 (-.11 to .15)
**Attitude towards justified litigation**						
Self-blame towards justified litigation (SBJL)			.12 (-.02 to .25)	-.03 (-.17 to .12)	-.06 (-.20 to .08)	-.06 (-.21 to .09)
Disapproval of justified litigation (DISJL)			.27 (.14 to .40)***	.25 (.12 to .37)***	.21 (.09 to .33)**	.21 (.08 to .33)**
**Attitude towards unjustified litigation**						
Concerns about unjustified litigation (COUJL)				.31 (.17 to .46)***	.28 (.14 to .42)***	.28 (.13 to .43)***
**Perceived patient pressure**						
Perceived patient pressure to refer (PPREF)					.18 (.03 to .33)*	.19 (.04 to .34)*
Perceived patient pressure to prescribe medicine (PPMED)					.23 (.09 to .36)**	.22 (.08 to .36)**
**Interactions**						
SBJL x PPREF						.02 (-.14 to .18)
SBJL x PPMED						.03 (-.14 to .19)
DISJL x PPREF						.00 (-.14 to .15)
DISJL x PPMED						-.03 (-.19 to .13)
COUJL x PPREF						-.03 (-.19 to .13)
COUJL x PPMED						.01 (-.17 to .16)

*** $P < 0.001$; ** $P < 0.01$; * $P < 0.05$;

For peer review only

For peer review only - http://bmjopen.bmj.com/site/about/guidelines.xhtml

Table 5

Results of regression analysis for avoidance behavior

Predictor	β (95% CI)					
	Model 1*** (R^2=.13)	Model 2*** (R^2=.13)	Model 3*** (R^2=.19)	Model 4*** (R^2=.21)	Model 5*** (R^2=.23)	Model 6*** (R^2=.24)
**Age > 60 (vs. age < 60)**	.26 (.12 to .39)***	.25 (.12 to .39)***	.28 (.15 to .41)***	.27 (.13 to .40)***	.27 (.14 to .40)***	.26 (.13 to .40)***
**Anesthesiology (vs. other specialisms)**	.24 (.10 to .37)**	.24 (.10 to .37)**	.23 (.10 to .37)**	.20 (.07 to .34)**	.21 (.07 to .34)**	.20 (.06 to .34)**
**Grade**						
Physician (vs. resident)		.01 (-.12 to .14)	.00 (-.13 to .13)	.00 (-.13 to .13)	-.01 (-.14 to .11)	.00 (-.14 to .12)
**Attitude towards justified litigation**						
Self-blame towards justified litigation (SBJL)			.15 (.02 to .28)*	.07 (-.08 to .21)	.07 (-.08 to .21)	.07 (-.08 to .22)
Disapproval of justified litigation (DISJL)			.17 (.04 to .30)**	.16 (.03 to .28)***	.16 (.03 to .28)*	.15 (.02 to .28)*
**Attitude towards unjustified litigation**						
Concerns about unjustified litigation (COUJL)				.18 (.04 to .33)***	.18 (.04 to .33)*	.17 (.01 to .32)*
**Perceived patient pressure**						
Perceived patient pressure to refer (PPREF)					-.04 (-.19 to .11)	-.04 (-.19 to .12)
Perceived patient pressure to prescribe medicine (PPMED)					.14 (.00 to .28)*	.12 (-.03 to .26)
**Interactions**						
SBJL x PPREF						-.04 (-.20 to .13)
SBJL x PPMED						.13 (-.04 to .30)
DISJL x PPREF						-.02 (-.17 to .14)
DISJL x PPMED						-.03 (-.19 to .14)
COUJL x PPREF						.01 (-.15 to .17)
COUJL x PPMED						-.01 (-.18 to .16)

*** $P < 0.001$; ** $P < 0.01$; * $P < 0.05$;

For peer review only - http://bmjopen.bmj.com/site/about/guidelines.xhtml

For peer review only

In line with hypothesis 1a, 'disapproval of justified litigation' was positively related to assurance behavior (*β*=.21, *p*<.01). However, no significant relationship was found between 'self-blame for justified litigation' and assurance behavior (*β*=-.06, *p*=.42). Turning to hypothesis 1b, 'disapproval of justified litigation' (*β*=.16, *p*<.05) was positively related to avoidance behavior, providing support for hypothesis 1b, whereas 'self-blame for justified litigation' was not (*β*=.07, *p*<.35). The negative attitudes towards justified litigation explained 9% of the variance in assurance behavior and 6% of the variance in avoidance behavior.

In hypothesis 2, it was suggested that a negative attitude towards unjustified litigation would be unrelated to a) assurance and b) avoidance behaviors. However, results showed that concerns about unjustified litigation were positively related to both assurance (*β*=.28, *p*<.001) and avoidance behaviors (*β*=.18, *p*<.05), leading us to reject hypothesis 2 (a and b). Additionally, a negative attitude towards unjustified litigation explained 8% of variance in assurance behavior and 2% of the variance in avoidance behavior.

Finally, the interaction between perceived patient pressure and negative litigation attitudes was tested in the prediction of assurance (hypothesis 3a) and avoidance (hypothesis 3b) behaviors. None of the interaction terms was significant. However, interestingly, perceived patient pressures to refer (*β*=.18, *p*<.05) and to prescribe medicine (β=.23, p<.01) had direct positive relationships with assurance behavior, explaining an additional 11% of the variance in assurance behavior. In terms of avoidance behavior, only perceived patient pressure to prescribe medicine was positively related to avoidance behavior (*β*=.14, *p*<.05).

For peer review only - http://bmjopen.bmj.com/site/about/guidelines.xhtml

Discussion and conclusions

The goal of this study was to investigate physicians' and residents' attitudes towards justified and unjustified litigation in relation to defensive medical behavior, and whether perceived patient pressures strengthen these relationships. Despite the low mean scores for assurance and avoidance behaviors, the vast majority of the respondents in this study did admit to engaging in assurance and avoidance behaviors (89% and 42% respectively). These findings of higher prevalence of assurance behavior are in line with previous studies.[4,6-11]

Those physicians and residents who disapproved of justified litigation, were more likely to employ assurance and avoidance behaviors. A possible explanation for their disapproval is the criminalization of errors, through which mistakes can be judged as negligence, which might lead to litigation.[43] No relationship was found between self-blame for justified litigation and physicians' defensive medical behavior. This was unexpected since previous research has found that the perception of personal failure following a complaint causes shame that induces physicians to adopt defensive medicine.[44] However, this study reveals that concerns about unjustified litigation seem to trigger avoidance and particularly assurance behaviors. It may be that physicians and residents associate unjustified litigation with stressful long-lasting litigation procedures[45] and negative media attention,[46] even if they are eventually cleared of any blame. This suggests that physicians and residents strongly fear becoming the 'second victim'[15,47,48] of an adverse event through unjustified litigation. This finding might also suggest that they fear being held accountable for what in their eyes is an unjustified litigation case, and this induces them to adopt defensive medical behaviors. If this is indeed the case, then a valuable step would be to find a way to prevent physicians and residents having to go through unjustified litigation

For peer review only - http://bmjopen.bmj.com/site/about/guidelines.xhtml

proceedings. This could perhaps be achieved by improving the filtering out of unjustified cases at an early stage to prevent them ending up in court.

Unlike in earlier studies,[10,11,28] this study did not reveal significant statistical differences between physicians and residents in either their attitudes towards litigation or their defensive medical behavior. This finding might be explained by a recent study[30] that reported that medical residents frequently encounter defensive medicine practices and are being taught to take malpractice liability into consideration when making clinical decisions. The findings regarding disapproval of justified litigation and concerns about unjustified litigation indicate which thoughts and emotions may be being passed on to residents during their practical training. As such, finding ways to prevent these thoughts and emotions being instilled during academic medical training is key to reducing defensive medical behavior.

It was further expected that perceived patient pressure would be a catalyst in the relationship between litigation attitude and defensive medical behavior. However, as no interaction effects were found, this catalytic role was not clearly evident. A possible reason for this is that both the means and the standard deviations of perceived patient pressures were low making it harder to detect a significant interaction[49]. Nevertheless, direct relationships between perceived patient pressure and both assurance and avoidance behaviors were identified. That is, physicians and residents who more often perceive pressure to refer or to prescribe medicine, more often demonstrate assurance behaviors than their colleagues who perceive these pressures less frequently. This finding may indicate that physicians and residents 'give in' to the demands of their patients in order to avoid conflicts, even if this goes against their professional judgement. Using assurance behaviors may be a way to prevent a good relationship with the patient going

For peer review only - http://bmjopen.bmj.com/site/about/guidelines.xhtml

sour. Indeed, the preservation of a good doctor-patient relationship has been suggested elsewhere as a potential explanation for defensive medical behavior.[9,50]

Although perceived pressure to prescribe medicine was related to avoidance behavior, it did not add significantly to the explained variance. This indicates that physicians and residents rarely respond to perceived patient pressures by refusing to treat patients or avoiding certain procedures, maybe because they do not see this as an appropriate response. The relationship that was found between perceived patient pressures and defensive medical behavior suggests that residents might be 'trained' to recognize patient pressures and accordingly act defensively. This would imply that it is important to provide training in the patient safety curriculum on communication skills to deal with perceived patient pressures and to not let these influence one's clinical decision-making. Physicians and residents should be made aware that their perceptions of patient pressures can lead them to adopt assurance and avoidance behaviors.

A strength of this study is that a broad spectrum of thoughts and emotions regarding litigation were measured, and a distinction was made between justified and unjustified types of litigation. Another strength of this study is that it is the first in which specific forms of patient pressure have been related to defensive medical behavior by physicians and residents, showing that perceptions of patient pressure are crucial in determining the adoption of defensive medical behaviors. Nevertheless, this study has several limitations. First, our data were self-reported perceptions and behaviors. Although there are no obvious concerns with the data in that variables are distinguishable and correlations between them are not excessive, other data sources could be used to verify the outcomes of this study. The second limitation is the study's cross-sectional design, which makes it impossible to determine the causality of relationships. Nevertheless, the literature [51,52] suggests that attitude is an important driver of behavior and supports the direction of the

For peer review only - http://bmjopen.bmj.com/site/about/guidelines.xhtml

relationships that were hypothesized between the theoretically linked variables. Future researchers are encouraged to use longitudinal studies to further investigate the relationships we found. Third, the research was executed in a context in which there is only a modest litigation climate. Given that behavior is context-specific, it is reasonable to expect the relationships found in this study for defensive medical behavior to be stronger in more litigious climates.

To summarize, this study demonstrates that physicians' and residents' 'disapproval of justified litigation' and their 'concerns about unjustified litigation' stimulate both assurance and avoidance forms of defensive medical behavior, whereas 'self-blame for justified litigation' did not. Further, perceptions of patient pressure to refer and to prescribe medicine stimulate physicians and residents to adopt both avoidance and assurance behaviors that can negatively affect the quality and safety of care. To prevent physicians implicitly instilling defensive medical behavior in residents, medical curricula should specifically address litigation attitudes and how to deal with perceived patient pressures.

Acknowledgements: The authors would like to thank all physicians and residents who participated in their study.

Contributors: ER designed the study, collected, analyzed and interpreted the data, and drafted the manuscript. KA codesigned the study, made significant contributions to the data acquisition and the interpretation of the data, and revised the manuscript. MB codesigned the study, made significant contributions to the analysis and interpretation of the data, and revised the manuscript. MT codesigned the study, contributed to the analysis and interpretation of the data, and revised the manuscript.

Competing interests: The authors declare that they have no competing interests.

For peer review only - http://bmjopen.bmj.com/site/about/guidelines.xhtml

Funding: This research received no specific grant from any funding agency in the public, commercial or not-for-profit sectors.

Data sharing statement: No additional data are available.

References

1 Kessler D, McClellan M. Do doctors practice defensive medicine? *Q J Econ.* 1996;111:353–90.

2 Dalton GD, Samaropoulos XF, Dalton AC. Effect of physician strategies for coping with the US medical malpractice crisis on healthcare delivery and patient access to healthcare. *Public Health.* 2008;122:1051–60.

3 DeKay LM, Asch DA. Is the use of diagnostic tests good for patients, or bad? *Med Decis Making.* 1998;18:19–28.

4 Panella M, Rinaldi C, Leigheb F, et al. Prevalence and cost of defensive medicine: a national survey of Italian physicians. *J Health Serv Res Policy.* 2017;0:1-7.

5 US Congress, Office of Technology Assessment. *Defensive Medicine and Medical Malpractice*. Pub. No. OTA-H-602. Washington, DC: US Government Printing Office; 1994.

6 Asher E, Dvir S, Seidman DS, et al., Defensive medicine among obstetricians and gynecologists in tertiary hospitals. *PloS one.* 2013;8:e57108.

7 Bishop TF, Federman AD, Keyhani S. Physicians' views on defensive medicine: a national survey. *Arch Intern Med.* 2010;170:1081–3.

8 Catino M. Blame culture and defensive medicine. *Cogn Technol Work.* 2009;11:245–53.

9 He AJ. The doctor–patient relationship, defensive medicine and overprescription in Chinese public hospitals: Evidence from a cross-sectional survey in Shenzhen city. *Soc Sci Med.* 2014;123:64–71.

For peer review only - http://bmjopen.bmj.com/site/about/guidelines.xhtml

10 Ortashi O, Virdee J, Hassan R, et al. The practice of defensive medicine among hospital doctors in the United Kingdom. *BMC Med Ethics.* 2013;14:42.

11 Studdert DM, Mello MM, Sage WM, et al. Defensive medicine among high-risk specialist physicians in a volatile malpractice environment. *JAMA.* 2005;293:2609–17.

12 Carrier ER, Reschovsky JD, Katz DA, et al. High physician concern about malpractice risk predicts more aggressive diagnostic testing in office-based practice. *Health Aff.* 2013;32:1383–91.

13 Nahed BV, Babu MA, Smith TR, et al. Malpractice liability and defensive medicine: a national survey of neurosurgeons. *PloS one.* 2012;7:e39237.

14 Nakamura N, Yamashita Y. Malpractice lawsuits and change in work in Japanese surgeons. *J Surg Res.* 2015;193:210–6.

15 Panella M, Rinaldi C, Leigheb F, et al. The determinants of defensive medicine in Italian hospitals: the impact of being a second victim. *Rev Calid Asist.* 2016;31;20-5.

16 Renkema E, Broekhuis MH, Ahaus K. Explaining the unexplainable–the impact of physicians' attitude towards litigation on their incident disclosure behaviour. *J Eval Clin Pract.* 2014;20:649–56.

17 Jena AB, Chandra A, Lakdawalla D, et al. Outcomes of medical malpractice litigation against US physicians. *Arch Intern Med.* 2012;172:892–4.

18 Peters Jr. PG. Twenty years of evidence on the outcomes of malpractice claims. *Clin Orthop Relat Res.* 2009;467:352–7.

19 Fletcher-Lartey S, Yee M, Gaarslev C, et al. Why do general practitioners prescribe antibiotics for upper respiratory tract infections to meet patient expectations: a mixed methods study. *BMJ Open.* 2016;6;e012244.

For peer review only - http://bmjopen.bmj.com/site/about/guidelines.xhtml

20 Lewis PJ, Tully MP. The discomfort caused by patient pressure on the prescribing decisions of hospital prescribers. *Res Social Adm Pharm.* 2011;7:4-15.

21 Little P, Dorward M, Warner G, et al. Importance of patient pressure and perceived pressure and perceived medical need for investigations, referral, and prescribing in primary care: nested observational study. *BMJ.* 2004;328:444.

22 Armstrong D, Fry J, Armstrong P. Doctors' perceptions of pressure from patients for referral. *BMJ.* 1991;302:1186–8.

23 Ringberg U, Fleten N, Førde OH. Examining the variation in GP's referral practice: a cross-sectional study of GP's reasons for referral. *Br J Gen Pract.* 2014;64:e426-33.

24 Fenton JJ, Franks P, Feldman MD, et al. Impact of patient requests on provider-perceived visit difficulty in primary care. *J Gen Intern Med.* 2015;30;214-20.

25 Ring A, Dowrick C, Humphris G, et al. Do patients with unexplained physical symptoms pressurise general practitioners for somatic treatment? A qualitative study. *BMJ.* 2004;328:1057.

26 Salmon P, Humphris GM, Ring A, et al. Why do primary care physicians propose medical care to patients with medically unexplained symptoms? A new method of sequence analysis to test theories of patient pressure. *Psychosom Med.* 2006;68:570–7.

27 Salmon P, Marchant-Haycox S. Surgery in the absence of pathology The relationship of patients' presentation to gynecologists' decisions for hysterectomy. *J Psychosom Res.* 2000;49:119–24.

28 Passmore K, Leung W-C. Defensive practice amongst psychiatrists: a questionnaire survey. *Postgrad Med J.* 2002;78:671–3.

29 Reed DA, Windish DM, Levine RB, et al. Do fears of malpractice litigation influence teaching behaviors? *Teach Learn Med.* 2008;20:205-11.

For peer review only - http://bmjopen.bmj.com/site/about/guidelines.xhtml

30 O'Leary KJ, Choi J, Watson K, et al. Medical students' and residents' clinical and educational experiences with defensive medicine. *Acad Med.* 2012;87:142-8.

31 Bark P, Vincent C, Olivieri L, et al. Impact of litigation on senior clinicians: implications for risk management. *Qual Saf Health Care.* 1997;6:7–13.

32 Benbassat J, Pilpel D, Schor R. Physicians' attitudes toward litigation and defensive practice: development of scale. *Behav Med.* 2001;27:52–60.

33 Bovbjerg RR, Miller RH, Shapiro DW. Paths to reducing medical injury: professional liability and discipline vs. patient safety – and the need for a third way. *J Law Med Ethics.* 2001;29:369–380.

34 De Winter JC, Dodou D. Factor recovery by principal axis factoring and maximum likelihood factor analysis as a function of factor pattern and sample size. *J Appl Stat.* 2012;39:695–710.

35 Kaiser HF. The application of electronic computers to factor analysis. *Educ Psychol Meas.* 1960:20:141–151.

36 Stevens JP. *Applied multivariate statistics for the social sciences* (fifth edition). New York, NY: Routledge; 2009.

37 Osborne JW, Costello AB. Best practices in exploratory factor analysis: Four recommendations for getting the most from your analysis. *Pan-Pacific Management Review* 2009;12:131–146.

38 Field A. *Discovering statistics using IBM SPSS statistics.* Thousand Oaks, CA: Sage; 2013.

39 Hayes, A.F. (2018). *Introduction to mediation, moderation, and conditional process analysis: A regression-based approach* (2nd Edition). New York, NY: Guilford Press; 2018.

40 Cole AP, Block L, Wu AW. On higher ground: ethical reasoning and its relationship with error disclosure. *BMJ Qual Saf.* 2013;22:580–5.

For peer review only - http://bmjopen.bmj.com/site/about/guidelines.xhtml

41 Durani P, Dias J, Singh HP, Taub N. Junior doctors and patient safety: evaluating knowledge, attitudes and perception of safety climate. *BMJ Qual Saf.* 2013;22:65–71.

42 Baruch Y, Holtom BC. Survey response rate levels and trends in organizational research. *Hum Relat.* 2008;61:1139–60.

43 Dekker S. *Just culture: Balancing safety and accountability.* Burlington, VT: Ashgate; 2007.

44 Cunningham W, Wilson H. Complaints, shame and defensive medicine. *BMJ Qual Saf.* 2011;20:449–52.

45 Seabury SA, Chandra A, Lakdwalla DN, et al. On average, physicians spend nearly 11 percent of their 40-year careers with an open, unresolved malpractice claim. *Health Aff* . 2013;32:111–9.

46 Dekker, Sidney. *Second victim: Error, guilt, trauma, and resilience.* Boca Raton, FL: CRC press; 2013.

47 Schiess C. Schwappach D, Schwendimann R, et al. A transactional ”second-victim” model–experiences of affected healthcare professionals in acute-somatic inpatient settings: a qualitative metasynthesis. *J Patient Saf.* 2018; DOI:10.1097/PTS.0000000000000461.

48 Scott SD, Hirschinger LE, Cox KR, et al. The natural history of recovery for the healthcare provider "second victim" after adverse patient events. *Qual Saf Health Care.* 2009;18:325–30.

49 Stone-Romero EF, Liakhovitski D. Strategies for detecting moderator variables: A review of conceptual and empirical issues. *Res Person Hum Resour Man.* 2002;21:333–72.

50 Cunningham W, Dovey S. Defensive changes in medical practice and the complaints process: a qualitative study of New Zealand doctors. *New Zeal Med J.* 2006;119.

51 Ajzen I. Nature and operation of attitudes. *Annu Rev Psychol.* 2001;52:27–58.

52 Loewenstein GF, Weber EU, Hsee CK, et al. Risk as feelings. *Psychol Bull.* 2001;127:267.

For peer review only - http://bmjopen.bmj.com/site/about/guidelines.xhtml

Appendix 1: Full survey instrument

	Strongly disagree	Disagree	Neutral	Agree	Strongly agree
Thoughts regarding potential individual consequences of malpractice litigation					
The potential consequences of <u>justified</u> accusations…					
1…are harmful for the reputation of a physician.	1	2	3	4	5
2…are bad for the self-confidence of a physician	1	2	3	4	5
3…are justified.	1	2	3	4	5
4…mean that physicians are being charged more often.	1	2	3	4	5
5…are a way to improve the quality of care.	1	2	3	4	5
The potential consequences of <u>unjustified</u> accusations…					
6…are harmful for the reputation of a physician.	1	2	3	4	5
7…are bad for the self-confidence of a physician	1	2	3	4	5
8…mean that physicians are being charged more often.	1	2	3	4	5
9…are an instrument to improve the quality of care.	1	2	3	4	5
Emotions from potential individual consequences of malpractice litigation					
The potential consequences of <u>justified</u> accusations…					
10…evoke in me the fear of being accused.	1	2	3	4	5
11…worry me.	1	2	3	4	5
12…make me feel angry.	1	2	3	4	5
13…make me feel stressed.	1	2	3	4	5
14…evoke a feeling of shame.	1	2	3	4	5
15…evoke a feeling of guilt.	1	2	3	4	5
16…I regard as a personal attack.	1	2	3	4	5
17…I regard as a form of justice.	1	2	3	4	5
The potential consequences of <u>unjustified</u> accusations…					
18…evoke in me the fear of being accused.	1	2	3	4	5
19…worry me.	1	2	3	4	5
20…make me feel angry.	1	2	3	4	5
21…make me feel stressed.	1	2	3	4	5
22…I regard as a personal attack.	1	2	3	4	5

For peer review only

For peer review only - http://bmjopen.bmj.com/site/about/guidelines.xhtml

	Never	Some-times	Fairly many times	Often	Always
Defensive medicine					
How often do you…					
23...order tests that are not clinically indicated in order to prevent a potential accusation?	1	2	3	4	5
24...carry out procedures that are probably unnecessary in order to prevent a potential accusation?	1	2	3	4	5
25...make unnecessary referrals to physicians with other specialisms to prevent a potential accusation?	1	2	3	4	5
26...prescribe unnecessary medication to prevent a potential accusation?	1	2	3	4	5
27...refuse to treat patients with complex medical problems in order to avoid a potential accusation in the event of a complication?	1	2	3	4	5
28...refuse to treat patients from which you expect an accusation in order to prevent such an accusation in the event of a complication?	1	2	3	4	5
29...avoid high-risk procedures in order to prevent a potential accusation in the event of a complication?	1	2	3	4	5
Perceived patient pressure to examine or execute treatment					
When a patient…					
30...describes their symptoms extensively in emotional words, I feel pressured to further examine or treat the patient even if it is clinically not strictly necessary.	1	2	3	4	5
31...directly or indirectly criticizes previous treatments, I feel pressured to further examine or treat the patient even if it is clinically not strictly necessary.	1	2	3	4	5
32...quotes decisions of other physicians to stress the need for intervention, I feel pressured to further examine or treat the patient even if it is clinically not strictly necessary.	1	2	3	4	5
33...requests further examination or treatment, I feel pressured to further examine or treat the patient even if it is clinically not strictly necessary.	1	2	3	4	5
Perceived patient pressure to refer the patient					
When a patient…					
34...describes their symptoms in extensive emotional words, I feel pressured to refer the patient even if it is clinically not strictly necessary.	1	2	3	4	5
35...directly or indirectly criticizes previous treatments, I feel pressured to refer the patient even if it is clinically not strictly necessary.	1	2	3	4	5
36...quotes decisions of other physicians to stress the need for intervention, I feel pressured to refer the patient even if it is clinically not strictly necessary.	1	2	3	4	5

For peer review only

37...requests further examination or treatment, I feel pressured to refer the patient even if it is clinically not strictly necessary.	1	2	3	4	5
Perceived patient pressure to prescribe medicine					
When a patient…					
38...describes their symptoms in extensive emotional words, I feel pressured to prescribe medicine even if it is clinically not strictly necessary.	1	2	3	4	5
39...directly or indirectly criticizes previous treatments, I feel pressured to prescribe medicine even if it is clinically not strictly necessary.	1	2	3	4	5
40...quotes decisions of other physicians to stress the need for intervention, I feel pressured to prescribe medicine even if it is clinically not strictly necessary.	1	2	3	4	5
41...requests further examination or treatment, I feel pressured to prescribe medicine even if it is clinically not strictly necessary.	1	2	3	4	5

For peer review only

STROBE 2007 (v4) Statement—Checklist of items that should be included in reports of *cross-sectional studies*

Checklist for manuscript 'Why is defensive behavior so deeply rooted in medicine? A cross-sectional study among physicians and residents in the Netherlands.

Section/Topic	Item #	Recommendation	Reported on page #
**Title and abstract**	1	(*a*) Indicate the study's design with a commonly used term in the title or the abstract	2
		(*b*) Provide in the abstract an informative and balanced summary of what was done and what was found	2
**Introduction**			
Background/rationale	2	Explain the scientific background and rationale for the investigation being reported	4, 5
Objectives	3	State specific objectives, including any prespecified hypotheses	4-6
**Methods**			
Study design	4	Present key elements of study design early in the paper	2, 6
Setting	5	Describe the setting, locations, and relevant dates, including periods of recruitment, exposure, follow-up, and data collection	2, 6
Participants	6	(*a*) Give the eligibility criteria, and the sources and methods of selection of participants	6, 7
Variables	7	Clearly define all outcomes, exposures, predictors, potential confounders, and effect modifiers. Give diagnostic criteria, if applicable	7-12
Data sources/ measurement	8*	For each variable of interest, give sources of data and details of methods of assessment (measurement). Describe comparability of assessment methods if there is more than one group	7-12
Bias	9	Describe any efforts to address potential sources of bias	6
Study size	10	Explain how the study size was arrived at	12-14
Quantitative variables	11	Explain how quantitative variables were handled in the analyses. If applicable, describe which groupings were chosen and why	8-12
Statistical methods	12	(*a*) Describe all statistical methods, including those used to control for confounding	8, 9
		(*b*) Describe any methods used to examine subgroups and interactions	8-12
		(*c*) Explain how missing data were addressed	8, 12-14
		(*d*) If applicable, describe analytical methods taking account of sampling strategy	12
		(*e*) Describe any sensitivity analyses	12

**Results**			
Participants	13*	(a) Report numbers of individuals at each stage of study—eg numbers potentially eligible, examined for eligibility, confirmed eligible, included in the study, completing follow-up, and analysed	12-14
		(b) Give reasons for non-participation at each stage	14
		(c) Consider use of a flow diagram	Not applicable
Descriptive data	14*	(a) Give characteristics of study participants (eg demographic, clinical, social) and information on exposures and potential confounders	13
		(b) Indicate number of participants with missing data for each variable of interest	8, 12-14
Outcome data	15*	Report numbers of outcome events or summary measures	15
Main results	16	(*a*) Give unadjusted estimates and, if applicable, confounder-adjusted estimates and their precision (eg, 95% confidence interval). Make clear which confounders were adjusted for and why they were included	17
		(*b*) Report category boundaries when continuous variables were categorized	17
		(*c*) If relevant, consider translating estimates of relative risk into absolute risk for a meaningful time period	Not applicable
Other analyses	17	Report other analyses done—eg analyses of subgroups and interactions, and sensitivity analyses	18
**Discussion**			
Key results	18	Summarise key results with reference to study objectives	20-22
Limitations	19	Discuss limitations of the study, taking into account sources of potential bias or imprecision. Discuss both direction and magnitude of any potential bias	20-22
Interpretation	20	Give a cautious overall interpretation of results considering objectives, limitations, multiplicity of analyses, results from similar studies, and other relevant evidence	20-22
Generalisability	21	Discuss the generalisability (external validity) of the study results	20-22
**Other information**			
Funding	22	Give the source of funding and the role of the funders for the present study and, if applicable, for the original study on which the present article is based	24

*Give information separately for cases and controls in case-control studies and, if applicable, for exposed and unexposed groups in cohort and cross-sectional studies.

Note: An Explanation and Elaboration article discusses each checklist item and gives methodological background and published examples of transparent reporting. The STROBE checklist is best used in conjunction with this article (freely available on the Web sites of PLoS Medicine at http://www.plosmedicine.org/, Annals of Internal Medicine at http://www.annals.org/, and Epidemiology at http://www.epidem.com/). Information on the STROBE Initiative is available at www.strobe-statement.org.

For peer review only

Triggers of defensive medical behaviors. A cross-sectional study among physicians in the Netherlands.

Journal:	*BMJ Open*
Manuscript ID	bmjopen-2018-025108.R2
Article Type:	Research
Date Submitted by the Author:	28-Feb-2019
Complete List of Authors:	Renkema, Erik; University of Groningen, Faculty of Economics and Business, Operations<br>Ahaus, Kees; University of Groningen, Faculty of Economics and Business, Operations<br>Broekhuis, Manda; University of Groningen, Faculty of Economics and Business, Operations<br>Tims, Maria; VU University Amsterdam, Faculty of Economics and Business Administration, Management & Organization
**Primary Subject Heading**:	Health services research
Secondary Subject Heading:	Patient-centred medicine, Ethics
Keywords:	Quality in health care < HEALTH SERVICES ADMINISTRATION & MANAGEMENT, Risk management < HEALTH SERVICES ADMINISTRATION & MANAGEMENT, MEDICAL ETHICS

SCHOLARONE™
Manuscripts

Triggers of defensive medical behaviors. A cross-sectional study among physicians in the Netherlands.

Authors: Erik Renkema, MSc, PhD, Kees Ahaus, MSc, PhD, Manda Broekhuis, MSc, PhD, Maria Tims, MSc, PhD.

E. Renkema is researcher, University of Groningen, Faculty of Economics and Business, Operations Department, Groningen, the Netherlands;

K. Ahaus is professor of healthcare management, University of Groningen, Faculty of Economics and Business, Operations Department, Groningen, the Netherlands.

M. Broekhuis is professor of professional service chains, University of Groningen, Faculty of Economics and Business, Operations Department, Groningen, the Netherlands.

M. Tims is associate professor, VU University Amsterdam, Faculty of Economics and Business Administration, Management & Organization Department, Amsterdam, the Netherlands;

Corresponding should be addressed to Erik Renkema, University of Groningen, Faculty of Economics and Business, Operations Department, P.O. Box 800, 9700 AV, Groningen, the Netherlands; telephone: +31-6-18864251; e-mail: e.h.renkema@rug.nl.

Keywords: litigation attitude, perceived patient pressure, defensive medicine

Word count: 3538

For peer review only - http://bmjopen.bmj.com/site/about/guidelines.xhtml

Abstract

Objectives This study investigated whether the attitudes of physicians towards justified and unjustified litigation, and their perception of patient pressure in demanding care, influence their use of defensive medical behaviors.

Design Cross-sectional survey using exploratory factor analysis to determine litigation attitude and perceived patient pressure factors. Regression analyses were used to regress these factors on to the ordering of extra tests or procedures (defensive assurance behavior) or the avoidance of high-risk patients or procedures (defensive avoidance behavior).

Setting Data were collected from eight Dutch hospitals.

Participants Respondents were 160 physicians and 54 residents (response rate 25%) of the hospital departments of (1) anesthesiology, (2) colon, stomach and liver diseases, (3) gynecology, (4) internal medicine, (5) neurology, and (6) surgery.

Primary outcome measures Respondents' application of defensive assurance and avoidance behaviors.

Results 'Disapproval of justified litigation' and 'Concerns about unjustified litigation' were positively related to both assurance (β=.21, $p<.01$, and β=.28, $p<.001$ respectively) and avoidance (β=.16, $p<.05$, and β=.18, $p<.05$ respectively) behaviors. 'Self-blame for justified litigation' was not significantly related to both defensive behaviors. Perceived patient pressures to refer (β=.18, $p<.05$) and to prescribe medicine (β=.23, $p<.01$) had direct positive relationships with assurance behavior, whereas perceived patient pressure to prescribe medicine was also positively related to avoidance behavior (β=.14, $p<.05$). No difference was found between physicians and residents in their defensive medical behavior.

Conclusions Physicians adopted more defensive medical behaviors if they had stronger thoughts and emotions towards (un)justified litigation. Further, physicians should be aware

For peer review only - http://bmjopen.bmj.com/site/about/guidelines.xhtml

that perceived patient pressure for care can lead to them adopting defensive behaviors that negatively affects the quality and safety of patient care.

Strengths and limitations of this study

- In this study a broad spectrum of thoughts and emotions regarding litigation were measured, and a distinction was made between justified and unjustified types of litigation.
- This is the first study in which specific forms of patient pressure have been related to physicians' and residents' defensive medical behavior.
- A drawback of the study is its reliance on self-reported perceptions and behavior.

For peer review only

For peer review only - http://bmjopen.bmj.com/site/about/guidelines.xhtml

Introduction

Defensive medicine is a widespread approach among physicians that negatively affects healthcare costs[1] and the quality and safety of patient care.[2,3] It is defined as the ordering of extra tests or procedures (assurance behavior), or the avoidance of high-risk patients or procedures (avoidance behavior), primarily to reduce the risk of being held liable for malpractice.[4,5] Studies show that between 60% and 95% of physicians adopt defensive medical behavior.[4,6-11] The inappropriate ordering of invasive procedures or medicines not only costs society unnecessary expense[4], it also exposes patients to unnecessary health risks[11]; while the avoidance of high-risk patients or procedures reduces patients' access to health care.[11] Traditionally, the adoption of defensive medical behavior is explained by concerns about malpractice litigation.[8,9,11-15] However, there is still considerable uncertainty as to how litigation concerns affect defensive medical behavior. For example, physicians vary in their thoughts and emotions towards litigation. Some regard litigation as a form of justice or as contributing to the quality of care, while others have a more negative attitude that is associated with emotions such as fear, stress, and anger, and a low self-confidence.[16] Despite these differences in how physicians think and feel about litigation and what they consider as justified and unjustified, the extent to which attitude differences play a role in defensive medical behavior is unknown. Furthermore, physicians' attitudes may differ for litigation which they view as 'justified', e.g. when they made a mistake or were negligent, and litigation which they view as 'unjustified', e.g. following an adverse event where they do not feel they acted incorrectly. A large proportion of malpractice litigation cases can be seen as 'unjustified' since they are dismissed in court[17] with physicians' behavior not being regarded as negligent.[18] The first objective of this study is to identify whether different attitudes towards justified and unjustified litigation lead to differences in defensive medical behavior.

For peer review only - http://bmjopen.bmj.com/site/about/guidelines.xhtml

This study focuses on thoughts and emotions of self-blame, concerns and disapproval which are for practical reasons labeled as 'negative', although in combination with litigation might reflect attitude aspects that align with the overall aims of the medical malpractice liability system.

Similarly, the aspect of patient pressure, in combination with litigation attitude, has not been addressed in defensive medicine research. Patients have become increasingly demanding in their requests for care, and may even pressurize physicians to provide specific forms of care.[19-21] Perceived pressure from patients is associated with higher referral rates,[22,23] and an increased likelihood of examining patients and over-prescribing drugs.[9,21,24-27] What remains unknown is whether perceived patient pressure strengthens the relationship between litigation attitude and defensive medicine. Consequently, the second objective of this study is to investigate whether perceived patient pressure drives physicians and residents to apply assurance or avoidance behaviors. Besides attending physicians (i.e. a person qualified to practice medicine), resident physicians (i.e. a medical graduate engaged in specialized practice under supervision in a hospital) were included in the study. Previous research[9,10,28] has found that defensive medicine is also prevalent amongst residents. Furthermore, some studies have reported that physicians alter their teaching behavior[29] to encourage their residents to practice defensively[30] in response to the fear of malpractice liability. As such, it seems important to investigate whether litigation attitude and patient pressure play a role in the defensive medical behaviors of residents. Based on this discussion, the following hypotheses have been formulated:

Hypothesis 1: A negative attitude towards justified litigation is positively related to a) assurance and b) avoidance behaviors by physicians and residents.

For peer review only - http://bmjopen.bmj.com/site/about/guidelines.xhtml

Hypothesis 2: A negative attitude towards unjustified litigation is unrelated to a) assurance and b) avoidance behaviors by physicians and residents.

Hypothesis 3: Perceived patient pressure for care strengthens the positive relationship between a negative attitude towards justified litigation and the a) assurance and b) avoidance behaviors by physicians and residents.

For peer review only

Method

Study design and sample

Between September 2014 and March 2015 a cross-sectional survey was conducted to investigate the relation between physicians' and residents' "negative" attitudes towards justified and unjustified litigation, their perception of patient pressure in demanding care, and their use of defensive medical behaviors.

Data were collected from physicians and residents working at eight Dutch hospitals. The hospitals are a mix of general and academic hospitals, geographically spread across the Netherlands. Prior to the data collection, the questionnaire was reviewed with one physician, and a pilot test carried out involving 32 physicians (data not included in the final analysis). The boards of directors, the medical staff or patient safety departments were asked for permission to distribute the questionnaire within their hospital in the departments of (1) anesthesiology, (2) colon, stomach and liver diseases, (3) gynecology, (4) internal medicine, (5) neurology, and (6) surgery. These specialisms were selected as they are relatively vulnerable to patients' complaints and litigation. After approval from the head of the medical staff of the relevant department(s), an email was sent to the physicians and residents containing a short explanation of the research and a hyperlink to the electronic survey. In the

For peer review only - http://bmjopen.bmj.com/site/about/guidelines.xhtml

email, and in the questionnaire, it was explicitly stated that all the information provided would be used anonymously. Participation was voluntary. Given that neither patients nor patients' data were involved in the study, ethical approval for the study was not required under the Dutch Law on Medical Research Involving Human Subjects (WMO).

Patient and public involvement

Patients and public were not involved in the development, design, recruitment and randomization of this study.

Measures

Negative attitudes towards justified and unjustified litigation were measured with 22 statements derived from existing research that probed thoughts and emotions regarding the potential individual consequences of justified and unjustified litigation[16,31-33]. Participants were asked about their emotions (e.g. guilt, worry, stress) and thoughts (e.g. improve the quality of care, a form of justice (reversed items)) about potential consequences of accusations. Participants were asked to respond to each statement using a five-point Likert scale ranging from 1 (strongly disagree) to 5 (strongly agree). See online supplementary appendix 1 for the full survey instrument.

Perceived patient pressure was measured with 12 statements derived from earlier studies on perceived patient pressure[21,25-27] that were adapted to match the practices of physicians. These statements were divided into three groups of four items, addressing perceived patient pressure to examine, to refer, and to prescribe medicine. Respondents rated how frequently they experienced patient pressure with options ranging from 1 (never) to 5 (always).

For peer review only - http://bmjopen.bmj.com/site/about/guidelines.xhtml

Defensive medicine was measured with 7 statements derived from studies on defensive medical behavior.[10,11] Participants were asked for assurance behaviors, such as prescribing unnecessary medication, ordering tests that are not clinically indicated, carrying out unnecessary procedures and making unnecessary referrals. They were also asked for avoidance behaviors, such as refusing to apply high-risk procedures. One previously used avoidance behavior statement (i.e. refusing to treat high-risk patients) was split into two separate statements (i.e. refusing to treat patients with complex medical problems, and refusing to treat patients from whom one expects an accusation) to add clarity. Response options again ranged from 1 (never) to 5 (always).

Data Analysis Strategy

Prior to testing the hypotheses, a factor analysis was conducted using SPSS Statistics 22 (IBM Corporation, Armonk, New York, USA) with Principal Axis Factoring[34] to examine the psychometric properties of the measures. The criterion for retaining factors was an eigenvalue[35] >1. Items were considered to contribute sufficiently to a factor when their loading[36] was >.36. Consequently, items that loaded <.36, or cross-loaded >.36, were removed during the analysis along with two-item factors since factors containing less than three items are usually weak.[37] To ease interpretation of the factor structure, we applied oblique rotation using direct oblimin and a delta of zero.[38] Cronbach's α values were calculated for the identified factors as well as the mean scores and standard deviations. Listwise deletion was applied in all analyses.

First, separate exploratory factor analyses were conducted on subsets of the data (i.e. litigation attitude, perceived patient pressure and defensive medical behavior) to determine the factor structure that best fitted each subset of items. Based on the separate factor analyses, 14 items were removed from the original dataset due to their low factor loadings, high cross-

For peer review only - http://bmjopen.bmj.com/site/about/guidelines.xhtml

loadings, or for being part of a two-item factor. Then the overall factor structure was checked through an exploratory factor analysis of the remaining 27 items (0.785; χ^2 (351) = 2255.868, p<.001). One further item was removed during this analysis due to a low factor loading and two more items that made up a two-item factor. Table 1 shows the seven-factor solution that emerged and the corresponding reliability estimates.

For peer review only

For peer review only - http://bmjopen.bmj.com/site/about/guidelines.xhtml

Table 1

Exploratory factor analysis (N=200), Cronbach's α values and factor loadings

Item wording	Loadings						
	SBJL	DISJL	COUJL	PPREF	PPMED	ASB	AVB
α	.726	.663	.860	.772	.884	.803	.650
**Self-blame towards justified litigation (SBJL)**							
The potential consequences of justified accusations evoke a feeling of guilt	**.903**	-.038	.015	.023	-.018	.036	.021
The potential consequences of justified accusations evoke a feeling of shame	**.760**	-.046	-.072	.029	-.004	.035	.096
I regard the potential consequences of justified accusations as a personal attack	**.372**	.111	-.057	-.136	-.018	-.092	-.195
**Disapproval of justified litigation (DISJL)**							
I regard the potential consequences of a justified accusation as a form of justice (reversed)	-.098	**.643**	-.016	-.126	.018	.094	.015
The potential consequences of justified accusations are justified (reversed)	-.064	**.612**	.018	.024	-.138	-.053	.019
The potential consequences of justified accusations are a way to improve the quality of care (reversed)	.054	**.561**	.098	.089	.131	.158	-.080
The potential consequences of justified accusations make me feel angry	.111	**.485**	-.117	-.045	.055	-.039	-.005
**Concerns about unjustified litigation (COUJL)**							
The potential consequences of unjustified accusations evoke in me the fear of being accused	-.022	-.028	**-.855**	.043	-.023	.057	.070
The potential consequences of unjustified accusations worry me	.028	-.040	**-.778**	.020	-.019	.048	-.106
The potential consequences of unjustified accusations make me feel stressed	.122	.090	**-.745**	-.070	.072	-.009	-.007
**Perceived patient pressure to refer (PPREF)**							
When a patient directly or indirectly criticizes previous treatments. I feel pressured to refer the patient even if it is clinically not strictly necessary	-.018	.029	-.037	**-.761**	.024	.036	-.044
When a patient quotes decisions of other physicians to stress the need for intervention. I feel pressured to refer the patient even if it is clinically not strictly necessary	.025	-.064	-.009	**-.743**	-.102	-.026	-.055
When a patient requests further examination or treatment. I feel pressured to refer the patient even if it is clinically not strictly necessary	.070	.126	.046	**-.546**	-.175	.069	.131

For peer review only

For peer review only - http://bmjopen.bmj.com/site/about/guidelines.xhtml

Table 1 (continued)

Exploratory factor analysis (N=200), Cronbach's α values and factor loadings

Item wording	Loadings						
	SBJL	DISJL	COULJ	PPREF	PPMED	ASB	AVB
α	.726	.663	.860	.772	.884	.803	.650
**Perceived patient pressure to prescribe medicine (PPMED)**							
When a patient describes their symptoms in extensive emotional words. I feel pressured to prescribe medicine even if it is clinically not strictly necessary	.022	.004	-.008	.055	**-.800**	-.035	-.089
When a patient quotes decisions of other physicians to stress the need for intervention. I feel pressured to prescribe medicine even if it is clinically not strictly necessary	-.020	.002	-.023	-.083	**-.789**	.087	.044
When a patient requests further examination or treatment. I feel pressured to prescribe medicine even if it is clinically not strictly necessary	-.013	.009	-.014	-.079	**-.785**	.027	.068
When a patient directly or indirectly criticizes previous treatments. I feel pressured to prescribe medicine even if it is clinically not strictly necessary	.040	-.036	.056	-.054	**-.764**	.092	-.060
**Assurance behavior (ASB)**							
How often do you prescribe unnecessary medication to prevent a potential accusation?	.039	.037	.098	.108	-.095	**.768**	.028
How often do you order tests that are not clinically indicated in order to prevent a potential accusation?	-.025	-.007	-.157	-.088	-.021	**.638**	-.058
How often do you carry out procedures that are probably unnecessary in order to prevent a potential accusation?	.017	.024	-.126	.008	-.063	**.626**	-.104
How often do you make unnecessary referrals to physicians with other specialisms to prevent a potential accusation?	-.016	.079	-.081	-.241	.028	**.595**	-.037
**Avoidance behavior (AVB)**							
How often do you refuse to treat patients with complex medical problems in order to avoid a potential accusation in the event of a complication	-.041	.108	-.093	.190	-.126	-.074	**-.659**
How often do you refuse to treat patients from whom you expect an accusation in order to prevent a potential accusation in the event of a complication	.019	-.068	.025	-.060	.081	.112	**-.604**
How often do you avoid high-risk procedures in order to prevent a potential accusation in the event of a complication	-.012	.013	-.002	-.092	-.040	.043	**-.592**
**Eigen values**	**1.516**	**2.235**	**1.098**	**1.250**	**3.124**	**5.295**	**1.771**
**% of variation explained (cumulative)**	**6.315**	**15.628**	**20.205**	**25.412**	**38.428**	**60.489**	**67.870**

For peer review only - http://bmjopen.bmj.com/site/about/guidelines.xhtml

In more detail, three factors relate to a physician's or resident's attitude towards litigation: self-blame for justified litigation (3 items); disapproval of justified litigation (4 items); and concerns about unjustified litigation (3 items). Two factors emerged related to perceived patient pressure: perceived patient pressure to refer (3 items); and perceived pressure to prescribe medicine (4 items).

Defensive medical behavior emerged as two distinct factors: assurance behavior (4 items); and avoidance behavior (3 items). The correlation between these two behaviors was low (r = .30; p<.01).

Each quantitative variable was summarized as a mean value and standard deviation. Next, the correlations between the control variables (gender, age, specialism, workplace, employment form, litigation experience, and formal measures after litigation) and the dependent variables (assurance and avoidance behaviors) were examined. This analysis showed that age (>60 years) and specialism (anesthesiology) correlated significantly with avoidance behavior and these were therefore included in the model as control variables. A dummy variable was included for 'grade' in the model to measure potential differences in defensive behavior between physicians and residents. Following this, the hypotheses were tested with two multiple regression analyses in which litigation attitude and perceived patient pressure were regressed on to assurance and avoidance behaviors. Finally, the interaction between litigation attitude and perceived patient pressure was investigated using a SPSS macro designed by Hayes.[39]

Results

A total of 238 responses were received. Due to missing data of 24 participants, the study sample consisted of 214 physicians and residents (response rate 25%), which is in line

For peer review only - http://bmjopen.bmj.com/site/about/guidelines.xhtml

with other studies regarding safety attitudes and medical incidents[40,41] and can be regarded as acceptable given the professionals approached.[42] The characteristics of the respondents are provided in Table 2. As shown in the second column, the sample is a good reflection of the target population.

Table 2

Characteristics of the survey participants (N=214) and target population[43]

Variables	No. of respondents (%)	Target Population (%)
**Gender**		
Male	121 (56.5)	5882 (55.0)
Female	93 (43.5)	4804 (45.0)
**Age**		
< 30	22 (10.3)	
30-40	68 (31.8)	
41-50	64 (29.9)	
51-60	50 (23.4)	
60<	10 (4.7)	
**Specialism**		
Anesthesiology	55 (25.7)	2307 (21.6)
Colon, stomach and liver	19 (8.9)	734 (6.9)
Gynecology	29 (13.6)	1436 (13.4)
Internal medicine	43 (20.1)	3148 (29.5)
Neurology	20 (9.3)	1295 (12.1)
Surgery	34 (15.9)	1766 (16.5)
Other	14 (6.5)	
**Grade**		
Physician	160 (74.8)	7936 (74.3)
Resident	54 (25.2)	2750 (25.7)
**Workplace**		
General hospital	132 (61.7)	
Academic hospital	78 (36.4)	
Other workplace	4 (1.9)	
**Employment form**		
Employed	119 (55.6)	
Self-employed	95 (44.4)	
**Litigation experience**		
Yes	34 (15.9)	
No	180 (84.1)	
**Formal measures after litigation (of N=34 with litigation experience)**		
Yes	4 (11.8)	
No	30 (88.2)	

For peer review only - http://bmjopen.bmj.com/site/about/guidelines.xhtml

Fourteen respondents were excluded from some of the analyses because they felt unable to answer some of the questions regarding defensive medicine and perceived patient pressure. This was because they regarded prescribing medicine as not a usual procedure within their specialism.

The means (standard deviations [SD]) of all variables and the correlations between the variables are displayed in Table 3. The analysis revealed that 177 of 200 respondents (89%) claimed to, at least sometimes, apply one or more forms of assurance behavior, and 90 of 214 respondents (42%) some form of avoidance behavior.

For peer review only - http://bmjopen.bmj.com/site/about/guidelines.xhtml

Table 3

Means, standard deviations and correlations among the study variables

Variable	M (SD)	1	2	3	4	5	6	7	8	9
1. Age (1 = over 60 years; 0 = 60 years or younger)	.05 (.21)									
2. Anesthesiologist (1 = Anest.; 0 = Other spec.)	.26 (.44)	.072								
3. Physician (1 = Physician; 0 = Resident)	.75 (.44)	.129	.071							
4. Self-blame towards justified litigation (SBJL)	3.31 (.76)	-.148*	-.064	-.030						
5. Disapproval of justified litigation (DISJL)	2.51 (.59)	-.011	.068	-.041	.092					
6. Concerns about unjustified litigation (COUJL)	3.98 (.77)	.025	.140*	.017	.439**	.139*				
7. Perceived patient pressure to refer (PPREF)	1.86 (.45)	.051	-.177**	.006	.223**	.154*	.215**			
8. Perceived patient pressure to prescribe medicine (PPMED)	1.58 (.48)	.016	-.060	.073	.040	.041	.038	.412**		
9. Assurance behavior (ASB)	1.76 (.57)	.072	.041	.057	.134	.286**	.332**	.350**	.315**	
10. Avoidance behavior AVB)	1.26 (.38)	.236**	.225**	.039	.104	.219**	.281**	.111	.131	.297**

** Significant at the 0.01 level (two-tailed); *Significant at the 0.05 level (two-tailed);

The means are the averages of participants' responses on the underlying items (range: 1-5)

For peer review only

Means for the perceived patient pressure factors were 1.86 (.45) and 1.58 (.48) respectively, and the individual items showed that 191 of 214 respondents (89%) felt, at least sometimes, pressured by their patients to refer, and 144 of 200 respondents (72%) to prescribe medicine. Perceived pressure to examine did not emerge as a distinct factor.

While most factors had an acceptable Cronbach's α (>.70), the factors related to disapproval of justified litigation and to avoidance behavior had reliability coefficients slightly below this threshold. The descriptive statistics show no signs of multicollinearity in the factor solution and support the assumption that all factors, except for self-blame for justified litigation, are significantly related to either assurance or avoidance behaviors. Factors identified in the exploratory factor analysis were entered into a multiple linear regression model and evaluated as possible predictors of both assurance and avoidance defensive medicine behavior.

Hypothesis testing

The regression analysis (Table 4 and Table 5) shows that the regression models that included litigation attitude and perceived patient pressures (model 5) were significant both for assurance behavior (F (13,186) = 5.817, p<.001) and for avoidance behavior (F (13,186) = 3.540, p<.001).

For peer review only

For peer review only - http://bmjopen.bmj.com/site/about/guidelines.xhtml

Table 4

Results of regression analysis for assurance behavior

Predictor	β (95% CI) Model 1 (R^2=.01)	Model 2 (R^2=.01)	Model 3** (R^2=.10)	Model 4*** (R^2=.18)	Model 5*** (R^2=.28)	Model 6*** (R^2=.29)
**Age > 60 (vs. age < 60)**	.07 (-.07 to .21)	.06 (-.08 to .21)	.09 (-.05 to .23)	.06 (-.07 to .20)	.05 (-.08 to .17)	.04 (-.09 to .17)
**Anesthesiology (vs. other specialisms)**	.04 (-.11 to .18)	.03 (-.12 to .18)	.02 (-.12 to .16)	-.03 (-.17 to .11)	.01 (-.12 to .14)	.01 (-.13 to .14)
**Grade**						
Physician (vs. resident)		.05 (-.10 to .19)	.04 (-.10 to .17)	.03 (-.10 to .16)	.02 (-.11 to .14)	.02 (-.11 to .15)
**Attitude towards justified litigation**						
Self-blame towards justified litigation (SBJL)			.12 (-.02 to .25)	-.03 (-.17 to .12)	-.06 (-.20 to .08)	-.06 (-.21 to .09)
Disapproval of justified litigation (DISJL)			.27 (.14 to .40)***	.25 (.12 to .37)***	.21 (.09 to .33)**	.21 (.08 to .33)**
**Attitude towards unjustified litigation**						
Concerns about unjustified litigation (COUJL)				.31 (.17 to .46)***	.28 (.14 to .42)***	.28 (.13 to .43)***
**Perceived patient pressure**						
Perceived patient pressure to refer (PPREF)					.18 (.03 to .33)*	.19 (.04 to .34)*
Perceived patient pressure to prescribe medicine (PPMED)					.23 (.09 to .36)**	.22 (.08 to .36)**
**Interactions**						
SBJL x PPREF						.02 (-.14 to .18)
SBJL x PPMED						.03 (-.14 to .19)
DISJL x PPREF						.00 (-.14 to .15)
DISJL x PPMED						-.03 (-.19 to .13)
COUJL x PPREF						-.03 (-.19 to .13)
COUJL x PPMED						.01 (-.17 to .16)

*** $P < 0.001$; ** $P < 0.01$; * $P < 0.05$;

For peer review only

Table 5

Results of regression analysis for avoidance behavior

Predictor	β (95% CI) Model 1*** (R^2=.13)	Model 2*** (R^2=.13)	Model 3*** (R^2=.19)	Model 4*** (R^2=.21)	Model 5*** (R^2=.23)	Model 6*** (R^2=.24)
**Age > 60 (vs. age < 60)**	.26 (.12 to .39)***	.25 (.12 to .39)***	.28 (.15 to .41)***	.27 (.13 to .40)***	.27 (.14 to .40)***	.26 (.13 to .40)***
**Anesthesiology (vs. other specialisms)**	.24 (.10 to .37)**	.24 (.10 to .37)**	.23 (.10 to .37)**	.20 (.07 to .34)**	.21 (.07 to .34)**	.20 (.06 to .34)**
**Grade**						
Physician (vs. resident)		.01 (-.12 to .14)	.00 (-.13 to .13)	.00 (-.13 to .13)	-.01 (-.14 to .11)	.00 (-.14 to .12)
**Attitude towards justified litigation**						
Self-blame towards justified litigation (SBJL)			.15 (.02 to .28)*	.07 (-.08 to .21)	.07 (-.08 to .21)	.07 (-.08 to .22)
Disapproval of justified litigation (DISJL)			.17 (.04 to .30)**	.16 (.03 to .28)***	.16 (.03 to .28)*	.15 (.02 to .28)*
**Attitude towards unjustified litigation**						
Concerns about unjustified litigation (COUJL)				.18 (.04 to .33)***	.18 (.04 to .33)*	.17 (.01 to .32)*
**Perceived patient pressure**						
Perceived patient pressure to refer (PPREF)					-.04 (-.19 to .11)	-.04 (-.19 to .12)
Perceived patient pressure to prescribe medicine (PPMED)					.14 (.00 to .28)*	.12 (-.03 to .26)
**Interactions**						
SBJL x PPREF						-.04 (-.20 to .13)
SBJL x PPMED						.13 (-.04 to .30)
DISJL x PPREF						-.02 (-.17 to .14)
DISJL x PPMED						-.03 (-.19 to .14)
COUJL x PPREF						.01 (-.15 to .17)
COUJL x PPMED						-.01 (-.18 to .16)

*** $P < 0.001$; ** $P < 0.01$; * $P < 0.05$;

For peer review only

For peer review only - http://bmjopen.bmj.com/site/about/guidelines.xhtml

In line with hypothesis 1a, 'disapproval of justified litigation' was positively related to assurance behavior (β=.21, p<.01). However, no significant relationship was found between 'self-blame for justified litigation' and assurance behavior (β=-.06, p=.42). Turning to hypothesis 1b, 'disapproval of justified litigation' (β=.16, p<.05) was positively related to avoidance behavior, providing support for hypothesis 1b, whereas 'self-blame for justified litigation' was not (β=.07, p<.35). The negative attitudes towards justified litigation explained 9% of the variance in assurance behavior and 6% of the variance in avoidance behavior.

In hypothesis 2, it was suggested that a negative attitude towards unjustified litigation would be unrelated to a) assurance and b) avoidance behaviors. However, results showed that concerns about unjustified litigation were positively related to both assurance (β=.28, p<.001) and avoidance behaviors (β=.18, p<.05), leading us to reject hypothesis 2 (a and b). Additionally, a negative attitude towards unjustified litigation explained 8% of variance in assurance behavior and 2% of the variance in avoidance behavior.

Finally, the interaction between perceived patient pressure and negative litigation attitudes was tested in the prediction of assurance (hypothesis 3a) and avoidance (hypothesis 3b) behaviors. None of the interaction terms was significant. However, interestingly, perceived patient pressures to refer (β=.18, p<.05) and to prescribe medicine (β=.23, p<.01) had direct positive relationships with assurance behavior, explaining an additional 11% of the variance in assurance behavior. In terms of avoidance behavior, only perceived patient pressure to prescribe medicine was positively related to avoidance behavior (β=.14, p<.05).

Discussion and conclusions

The goal of this study was to investigate physicians' and residents' attitudes towards justified and unjustified litigation in relation to defensive medical behavior, and whether perceived

For peer review only - http://bmjopen.bmj.com/site/about/guidelines.xhtml

patient pressures strengthen these relationships. Despite the low mean scores for assurance and avoidance behaviors, the vast majority of the respondents in this study did admit to engaging in assurance and avoidance behaviors (89% and 42% respectively). These findings of higher prevalence of assurance behavior are in line with previous studies.[4,6-11]

Those physicians and residents who disapproved of justified litigation, were more likely to employ assurance and avoidance behaviors. A possible explanation for their disapproval is the criminalization of errors, through which mistakes can be judged as negligence, which might lead to litigation.[44] No relationship was found between self-blame for justified litigation and physicians' defensive medical behavior. This was unexpected since previous research has found that the perception of personal failure following a complaint causes shame that induces physicians to adopt defensive medicine.[45] However, this study reveals that concerns about unjustified litigation seem to trigger avoidance and particularly assurance behaviors. It may be that physicians and residents associate unjustified litigation with stressful long-lasting litigation procedures[46] and negative media attention,[47] even if they are eventually cleared of any blame. This suggests that physicians and residents strongly fear becoming the 'second victim'[15,48,49] of an adverse event through unjustified litigation. This finding might also suggest that they fear being held accountable for what in their eyes is an unjustified litigation case, and this induces them to adopt defensive medical behaviors. If this is indeed the case, then a valuable step would be to find a way to prevent physicians and residents having to go through unjustified litigation proceedings. This could perhaps be achieved by improving the filtering out of unjustified cases at an early stage to prevent them ending up in court.

Unlike in earlier studies,[10,11,28] this study did not reveal significant statistical differences between physicians and residents in either their negative attitudes towards litigation or their

For peer review only - http://bmjopen.bmj.com/site/about/guidelines.xhtml

defensive medical behavior. This finding might be explained by a recent study[30] that reported that medical residents frequently encounter defensive medicine practices and are being taught to take malpractice liability into consideration when making clinical decisions. The findings regarding disapproval of justified litigation and concerns about unjustified litigation indicate which thoughts and emotions may be being passed on to residents during their practical training. As such, finding ways to prevent these thoughts and emotions being instilled during academic medical training is key to reducing defensive medical behavior.

It was further expected that perceived patient pressure would be a catalyst in the relationship between litigation attitude and defensive medical behavior. However, as no interaction effects were found, this catalytic role was not clearly evident. A possible reason for this is that both the means and the standard deviations of perceived patient pressures were low making it harder to detect a significant interaction[50]. Nevertheless, direct relationships between perceived patient pressure and both assurance and avoidance behaviors were identified. That is, physicians and residents who more often perceive pressure to refer or to prescribe medicine, more often demonstrate assurance behaviors than their colleagues who perceive these pressures less frequently. This finding may indicate that physicians and residents 'give in' to the demands of their patients in order to avoid conflicts, even if this goes against their professional judgement. Using assurance behaviors may be a way to prevent a good relationship with the patient going sour. Indeed, the preservation of a good doctor-patient relationship has been suggested elsewhere as a potential explanation for defensive medical behavior.[9,51]

Although perceived pressure to prescribe medicine was related to avoidance behavior, it did not add significantly to the explained variance. This indicates that physicians and residents rarely respond to perceived patient pressures by refusing to treat patients or avoiding

For peer review only - http://bmjopen.bmj.com/site/about/guidelines.xhtml

certain procedures, maybe because they do not see this as an appropriate response. The relationship that was found between perceived patient pressures and defensive medical behavior suggests that residents might be 'trained' to recognize patient pressures and accordingly act defensively. This would imply that it is important to provide training in the patient safety curriculum on communication skills to deal with perceived patient pressures and to not let these influence one's clinical decision-making. Physicians and residents should be made aware that their perceptions of patient pressures can lead them to adopt assurance and avoidance behaviors.

For respondents older than sixty years, there was a significant relationship with avoidance behavior. An explanation for this finding may be found in the Selection Optimization Theory.[52, 53] This theory states that people, when they are older, prefer to undertake less complicated and risky tasks than younger people. Our findings are in line with a study by Marin[54], showing more defensive practices amongst older doctors. Though some scholars found that younger physicians show more defensive medicine behavior.[10] Therefore more research is needed to conclude on the relationship between age and defensive medicine behavior.

Anesthesiologists were more inclined to refuse high-risk patients and procedures than the other specialists we included in our study. With its longstanding and strong focus on reducing patient harm, anesthesiology is seen as the specialism that provides the standard for patient safety.[55] Therefore, an explanation for our finding might be that the strong focus on reducing patient harm has translated into a greater emphasis on risk-averse behavior by anesthesiologists than by those in other specialisms. Future research is needed to see whether this relationship is confirmed elsewhere.

For peer review only - http://bmjopen.bmj.com/site/about/guidelines.xhtml

A strength of this study is that a broad spectrum of thoughts and emotions regarding litigation were measured, and a distinction was made between justified and unjustified types of litigation. Another strength of this study is that it is the first in which specific forms of patient pressure have been related to defensive medical behavior by physicians and residents, showing that perceptions of patient pressure are crucial in determining the adoption of defensive medical behaviors. A third strength is that we were able to collect the responses of 200 physicians and residents on the delicate topic of defensive behaviors, and that this sample turned out to be an adequate representation of the targeted population. Nevertheless, this study has several limitations. First, our data were self-reported perceptions and behaviors. Although there are no obvious concerns with the data in that variables are distinguishable and correlations between them are not excessive, other data sources could be used to verify the outcomes of this study. The second limitation is the study's cross-sectional design, which makes it impossible to determine the causality of relationships. Nevertheless, the literature [56,57] suggests that attitude is an important driver of behavior and supports the direction of the relationships that were hypothesized between the theoretically linked variables. Future researchers are encouraged to use longitudinal studies to further investigate the relationships we found. Third, the research was executed in a context in which there is a relatively modest litigation climate. Given that behavior is context-specific, it is reasonable to expect the relationships found in this study for defensive medical behavior to be stronger in more litigious climates.

Another promising area for future research are care settings outside of hospitals. As general practitioners often refer patients to hospitals for further investigation, primary care might for example be an interesting setting to investigate the relationships we found.

For peer review only - http://bmjopen.bmj.com/site/about/guidelines.xhtml

To summarize, this study demonstrates that physicians' and residents' 'disapproval of justified litigation' and their 'concerns about unjustified litigation' stimulate both assurance and avoidance forms of defensive medical behavior, whereas 'self-blame for justified litigation' did not. Further, perceptions of patient pressure to refer and to prescribe medicine stimulate physicians and residents to adopt both avoidance and assurance behaviors that can negatively affect the quality and safety of care. To prevent physicians implicitly instilling defensive medical behavior in residents, medical curricula should specifically address litigation attitudes and how to deal with perceived patient pressures.

Acknowledgements: The authors would like to thank all physicians and residents who participated in their study.

Contributors: ER designed the study, collected, analyzed and interpreted the data, and drafted the manuscript. KA co-designed the study, made significant contributions to the data acquisition and the interpretation of the data, and revised the manuscript. MB co-designed the study, made significant contributions to the analysis and interpretation of the data, and revised the manuscript. MT co-designed the study, contributed to the analysis and interpretation of the data, and revised the manuscript.

Competing interests: The authors declare that they have no competing interests.

Funding: This research received no specific grant from any funding agency in the public, commercial or not-for-profit sectors.

Data sharing statement: No additional data are available.

References

1 Kessler D, McClellan M. Do doctors practice defensive medicine? *Q J Econ.* 1996;111:353–90.

For peer review only - http://bmjopen.bmj.com/site/about/guidelines.xhtml

2 Dalton GD, Samaropoulos XF, Dalton AC. Effect of physician strategies for coping with the US medical malpractice crisis on healthcare delivery and patient access to healthcare. *Public Health.* 2008;122:1051–60.

3 DeKay LM, Asch DA. Is the use of diagnostic tests good for patients, or bad? *Med Decis Making.* 1998;18:19–28.

4 Panella M, Rinaldi C, Leigheb F, et al. Prevalence and cost of defensive medicine: a national survey of Italian physicians. *J Health Serv Res Policy.* 2017;0:1-7.

5 US Congress, Office of Technology Assessment. *Defensive Medicine and Medical Malpractice*. Pub. No. OTA-H-602. Washington, DC: US Government Printing Office; 1994.

6 Asher E, Dvir S, Seidman DS, et al., Defensive medicine among obstetricians and gynecologists in tertiary hospitals. *PloS one.* 2013;8:e57108.

7 Bishop TF, Federman AD, Keyhani S. Physicians' views on defensive medicine: a national survey. *Arch Intern Med.* 2010;170:1081–3.

8 Catino M. Blame culture and defensive medicine. *Cogn Technol Work.* 2009;11:245–53.

9 He AJ. The doctor–patient relationship, defensive medicine and overprescription in Chinese public hospitals: Evidence from a cross-sectional survey in Shenzhen city. *Soc Sci Med.* 2014;123:64–71.

10 Ortashi O, Virdee J, Hassan R, et al. The practice of defensive medicine among hospital doctors in the United Kingdom. *BMC Med Ethics.* 2013;14:42.

11 Studdert DM, Mello MM, Sage WM, et al. Defensive medicine among high-risk specialist physicians in a volatile malpractice environment. *JAMA.* 2005;293:2609–17.

12 Carrier ER, Reschovsky JD, Katz DA, et al. High physician concern about malpractice risk predicts more aggressive diagnostic testing in office-based practice. *Health Aff.* 2013;32:1383–91.

For peer review only - http://bmjopen.bmj.com/site/about/guidelines.xhtml

13 Nahed BV, Babu MA, Smith TR, et al. Malpractice liability and defensive medicine: a national survey of neurosurgeons. *PloS one.* 2012;7:e39237.

14 Nakamura N, Yamashita Y. Malpractice lawsuits and change in work in Japanese surgeons. *J Surg Res.* 2015;193:210–6.

15 Panella M, Rinaldi C, Leigheb F, et al. The determinants of defensive medicine in Italian hospitals: the impact of being a second victim. *Rev Calid Asist.* 2016;31;20-5.

16 Renkema E, Broekhuis MH, Ahaus K. Explaining the unexplainable–the impact of physicians' attitude towards litigation on their incident disclosure behaviour. *J Eval Clin Pract.* 2014;20:649–56.

17 Jena AB, Chandra A, Lakdawalla D, et al. Outcomes of medical malpractice litigation against US physicians. *Arch Intern Med.* 2012;172:892–4.

18 Peters Jr. PG. Twenty years of evidence on the outcomes of malpractice claims. *Clin Orthop Relat Res.* 2009;467:352–7.

19 Fletcher-Lartey S, Yee M, Gaarslev C, et al. Why do general practitioners prescribe antibiotics for upper respiratory tract infections to meet patient expectations: a mixed methods study. *BMJ Open.* 2016;6;e012244.

20 Lewis PJ, Tully MP. The discomfort caused by patient pressure on the prescribing decisions of hospital prescribers. *Res Social Adm Pharm.* 2011;7:4-15.

21 Little P, Dorward M, Warner G, et al. Importance of patient pressure and perceived pressure and perceived medical need for investigations, referral, and prescribing in primary care: nested observational study. *BMJ.* 2004;328:444.

22 Armstrong D, Fry J, Armstrong P. Doctors' perceptions of pressure from patients for referral. *BMJ.* 1991;302:1186–8.

23 Ringberg U, Fleten N, Førde OH. Examining the variation in GP's referral practice: a cross-sectional study of GP's reasons for referral. *Br J Gen Pract.* 2014;64:e426-33.

For peer review only - http://bmjopen.bmj.com/site/about/guidelines.xhtml

24 Fenton JJ, Franks P, Feldman MD, et al. Impact of patient requests on provider-perceived visit difficulty in primary care. *J Gen Intern Med.* 2015;30;214-20.

25 Ring A, Dowrick C, Humphris G, et al. Do patients with unexplained physical symptoms pressurise general practitioners for somatic treatment? A qualitative study. *BMJ.* 2004;328:1057.

26 Salmon P, Humphris GM, Ring A, et al. Why do primary care physicians propose medical care to patients with medically unexplained symptoms? A new method of sequence analysis to test theories of patient pressure. *Psychosom Med.* 2006;68:570–7.

27 Salmon P, Marchant-Haycox S. Surgery in the absence of pathology The relationship of patients' presentation to gynecologists' decisions for hysterectomy. *J Psychosom Res.* 2000;49:119–24.

28 Passmore K, Leung W-C. Defensive practice amongst psychiatrists: a questionnaire survey. *Postgrad Med J.* 2002;78:671–3.

29 Reed DA, Windish DM, Levine RB, et al. Do fears of malpractice litigation influence teaching behaviors? *Teach Learn Med.* 2008;20:205-11.

30 O'Leary KJ, Choi J, Watson K, et al. Medical students' and residents' clinical and educational experiences with defensive medicine. *Acad Med.* 2012;87:142-8.

31 Bark P, Vincent C, Olivieri L, et al. Impact of litigation on senior clinicians: implications for risk management. *Qual Saf Health Care.* 1997;6:7–13.

32 Benbassat J, Pilpel D, Schor R. Physicians' attitudes toward litigation and defensive practice: development of scale. *Behav Med.* 2001;27:52–60.

33 Bovbjerg RR, Miller RH, Shapiro DW. Paths to reducing medical injury: professional liability and discipline vs. patient safety – and the need for a third way. *J Law Med Ethics.* 2001;29:369–380.

For peer review only - http://bmjopen.bmj.com/site/about/guidelines.xhtml

34 De Winter JC, Dodou D. Factor recovery by principal axis factoring and maximum likelihood factor analysis as a function of factor pattern and sample size. *J Appl Stat.* 2012;39:695–710.

35 Kaiser HF. The application of electronic computers to factor analysis. *Educ Psychol Meas.* 1960:20:141–151.

36 Stevens JP. *Applied multivariate statistics for the social sciences* (fifth edition). New York, NY: Routledge; 2009.

37 Osborne JW, Costello AB. Best practices in exploratory factor analysis: Four recommendations for getting the most from your analysis. *Pan-Pacific Management Review* 2009;12:131–146.

38 Field A. *Discovering statistics using IBM SPSS statistics.* Thousand Oaks, CA: Sage; 2013.

39 Hayes, A.F. (2018). *Introduction to mediation, moderation, and conditional process analysis: A regression-based approach* (2nd Edition). New York, NY: Guilford Press; 2018.

40 Cole AP, Block L, Wu AW. On higher ground: ethical reasoning and its relationship with error disclosure. *BMJ Qual Saf.* 2013;22:580–5.

41 Durani P, Dias J, Singh HP, Taub N. Junior doctors and patient safety: evaluating knowledge, attitudes and perception of safety climate. *BMJ Qual Saf.* 2013;22:65–71.

42 Baruch Y, Holtom BC. Survey response rate levels and trends in organizational research. *Hum Relat.* 2008;61:1139–60.

43 Stichting Capaciteitsorgaan voor Medische en Tandheelkundige Vervolgopleidingen. *Capaciteitsplan 2016 Deelrapport 1*. Utrecht. Stichting Capaciteitsorgaan voor Medische en Tandheelkundige Vervolgopleidingen 2016.

For peer review only - http://bmjopen.bmj.com/site/about/guidelines.xhtml

44 Dekker S. *Just culture: Balancing safety and accountability.* Burlington, VT: Ashgate; 2007.

45 Cunningham W, Wilson H. Complaints, shame and defensive medicine. *BMJ Qual Saf.* 2011;20:449–52.

46 Seabury SA, Chandra A, Lakdwalla DN, et al. On average, physicians spend nearly 11 percent of their 40-year careers with an open, unresolved malpractice claim. *Health Aff* . 2013;32:111–9.

47 Dekker, S. *Second victim: Error, guilt, trauma, and resilience.* Boca Raton, FL: CRC press; 2013.

48 Schiess C, Schwappach D, Schwendimann R, et al. A transactional ”second-victim” model–experiences of affected healthcare professionals in acute-somatic inpatient settings: a qualitative metasynthesis. *J Patient Saf.* 2018; DOI:10.1097/PTS.0000000000000461.

49 Scott SD, Hirschinger LE, Cox KR, et al. The natural history of recovery for the healthcare provider “second victim” after adverse patient events. *Qual Saf Health Care.* 2009;18:325–30.

50 Stone-Romero EF, Liakhovitski D. Strategies for detecting moderator variables: A review of conceptual and empirical issues. *Res Person Hum Resour Man.* 2002;21:333–72.

51 Cunningham W, Dovey S. Defensive changes in medical practice and the complaints process: a qualitative study of New Zealand doctors. *New Zeal Med J.* 2006;119.

52 Baltes PB, Baltes MM. Psychological perspectives on successful aging: The model of selective optimization with compensation. In Baltes PB, Baltes MM eds. Successful aging: Perspectives from the behavioral sciences. New York: NY. Cambridge University Press 1990:1-34.

For peer review only - http://bmjopen.bmj.com/site/about/guidelines.xhtml

53 Baltes PB. On the incomplete architecture of human ontogeny: Selection, optimization, and compensation as foundation of developmental theory. *Am Psychol*. 1997;52:366-80.

54 Marin PP, Bayer AJ, Tomlinson A, et al. Attitudes of hospital doctors in Wales to use of intravenous fluids and antibiotics in the terminally ill. *Postgrad Med J.* 1989;65:650-2.

55 Schnittker R, Marshall SD. Safe anaesthetic care: further improvements require a focus on resilience. *Br J Anaesth* 2015;115:643–5.

56 Ajzen I. Nature and operation of attitudes. *Annu Rev Psychol.* 2001;52:27–58.

57 Loewenstein GF, Weber EU, Hsee CK, et al. Risk as feelings. *Psychol Bull.* 2001;127:267.

For peer review only

Appendix 1: Full survey instrument

	Strongly disagree	Disagree	Neutral	Agree	Strongly agree
Thoughts regarding potential individual consequences of malpractice litigation					
The potential consequences of justified accusations…					
1…are harmful for the reputation of a physician.	1	2	3	4	5
2…are bad for the self-confidence of a physician	1	2	3	4	5
3…are justified.	1	2	3	4	5
4…mean that physicians are being charged more often.	1	2	3	4	5
5…are a way to improve the quality of care.	1	2	3	4	5
The potential consequences of unjustified accusations…					
6…are harmful for the reputation of a physician.	1	2	3	4	5
7…are bad for the self-confidence of a physician	1	2	3	4	5
8…mean that physicians are being charged more often.	1	2	3	4	5
9…are an instrument to improve the quality of care.	1	2	3	4	5
Emotions from potential individual consequences of malpractice litigation					
The potential consequences of justified accusations…					
10…evoke in me the fear of being accused.	1	2	3	4	5
11…worry me.	1	2	3	4	5
12…make me feel angry.	1	2	3	4	5
13…make me feel stressed.	1	2	3	4	5
14…evoke a feeling of shame.	1	2	3	4	5
15…evoke a feeling of guilt.	1	2	3	4	5
16…I regard as a personal attack.	1	2	3	4	5
17…I regard as a form of justice.	1	2	3	4	5
The potential consequences of unjustified accusations…					
18…evoke in me the fear of being accused.	1	2	3	4	5
19…worry me.	1	2	3	4	5
20…make me feel angry.	1	2	3	4	5
21…make me feel stressed.	1	2	3	4	5
22…I regard as a personal attack.	1	2	3	4	5

For peer review only - http://bmjopen.bmj.com/site/about/guidelines.xhtml

	Never	Some-times	Fairly many times	Often	Always
Defensive medicine					
How often do you…					
23...order tests that are not clinically indicated in order to prevent a potential accusation?	1	2	3	4	5
24...carry out procedures that are probably unnecessary in order to prevent a potential accusation?	1	2	3	4	5
25...make unnecessary referrals to physicians with other specialisms to prevent a potential accusation?	1	2	3	4	5
26...prescribe unnecessary medication to prevent a potential accusation?	1	2	3	4	5
27...refuse to treat patients with complex medical problems in order to avoid a potential accusation in the event of a complication?	1	2	3	4	5
28...refuse to treat patients from which you expect an accusation in order to prevent such an accusation in the event of a complication?	1	2	3	4	5
29...avoid high-risk procedures in order to prevent a potential accusation in the event of a complication?	1	2	3	4	5
Perceived patient pressure to examine or execute treatment					
When a patient…					
30...describes their symptoms extensively in emotional words, I feel pressured to further examine or treat the patient even if it is clinically not strictly necessary.	1	2	3	4	5
31...directly or indirectly criticizes previous treatments, I feel pressured to further examine or treat the patient even if it is clinically not strictly necessary.	1	2	3	4	5
32...quotes decisions of other physicians to stress the need for intervention, I feel pressured to further examine or treat the patient even if it is clinically not strictly necessary.	1	2	3	4	5
33...requests further examination or treatment, I feel pressured to further examine or treat the patient even if it is clinically not strictly necessary.	1	2	3	4	5
Perceived patient pressure to refer the patient					
When a patient…					
34...describes their symptoms in extensive emotional words, I feel pressured to refer the patient even if it is clinically not strictly necessary.	1	2	3	4	5
35...directly or indirectly criticizes previous treatments, I feel pressured to refer the patient even if it is clinically not strictly necessary.	1	2	3	4	5
36...quotes decisions of other physicians to stress the need for intervention, I feel pressured to refer the patient even if it is clinically not strictly necessary.	1	2	3	4	5

For peer review only - http://bmjopen.bmj.com/site/about/guidelines.xhtml

37...requests further examination or treatment, I feel pressured to refer the patient even if it is clinically not strictly necessary.	1	2	3	4	5
Perceived patient pressure to prescribe medicine					
When a patient…					
38...describes their symptoms in extensive emotional words, I feel pressured to prescribe medicine even if it is clinically not strictly necessary.	1	2	3	4	5
39...directly or indirectly criticizes previous treatments, I feel pressured to prescribe medicine even if it is clinically not strictly necessary.	1	2	3	4	5
40...quotes decisions of other physicians to stress the need for intervention, I feel pressured to prescribe medicine even if it is clinically not strictly necessary.	1	2	3	4	5
41...requests further examination or treatment, I feel pressured to prescribe medicine even if it is clinically not strictly necessary.	1	2	3	4	5

For peer review only

STROBE 2007 (v4) Statement—Checklist of items that should be included in reports of *cross-sectional studies*

Checklist for manuscript 'Triggers of defensive medical behaviors. A cross-sectional study among physicians in the Netherlands.

Section/Topic	Item #	Recommendation	Reported on page #
**Title and abstract**	1	(*a*) Indicate the study's design with a commonly used term in the title or the abstract	2
		(*b*) Provide in the abstract an informative and balanced summary of what was done and what was found	2
**Introduction**			
Background/rationale	2	Explain the scientific background and rationale for the investigation being reported	4, 5
Objectives	3	State specific objectives, including any prespecified hypotheses	4-6
**Methods**			
Study design	4	Present key elements of study design early in the paper	2, 6
Setting	5	Describe the setting, locations, and relevant dates, including periods of recruitment, exposure, follow-up, and data collection	2, 6, 7
Participants	6	(*a*) Give the eligibility criteria, and the sources and methods of selection of participants	6, 7
Variables	7	Clearly define all outcomes, exposures, predictors, potential confounders, and effect modifiers. Give diagnostic criteria, if applicable	7-12
Data sources/ measurement	8*	For each variable of interest, give sources of data and details of methods of assessment (measurement). Describe comparability of assessment methods if there is more than one group	7-12
Bias	9	Describe any efforts to address potential sources of bias	6
Study size	10	Explain how the study size was arrived at	12-14
Quantitative variables	11	Explain how quantitative variables were handled in the analyses. If applicable, describe which groupings were chosen and why	8-13
Statistical methods	12	(*a*) Describe all statistical methods, including those used to control for confounding	8, 9
		(*b*) Describe any methods used to examine subgroups and interactions	8-13
		(*c*) Explain how missing data were addressed	8, 12-14
		(*d*) If applicable, describe analytical methods taking account of sampling strategy	13
		(*e*) Describe any sensitivity analyses	13

For peer review only

**Results**			
Participants	13*	(a) Report numbers of individuals at each stage of study—eg numbers potentially eligible, examined for eligibility, confirmed eligible, included in the study, completing follow-up, and analysed	12-14
		(b) Give reasons for non-participation at each stage	14
		(c) Consider use of a flow diagram	Not applicable
Descriptive data	14*	(a) Give characteristics of study participants (eg demographic, clinical, social) and information on exposures and potential confounders	13
		(b) Indicate number of participants with missing data for each variable of interest	8, 12-14
Outcome data	15*	Report numbers of outcome events or summary measures	16
Main results	16	(*a*) Give unadjusted estimates and, if applicable, confounder-adjusted estimates and their precision (eg, 95% confidence interval). Make clear which confounders were adjusted for and why they were included	17
		(*b*) Report category boundaries when continuous variables were categorized	17
		(*c*) If relevant, consider translating estimates of relative risk into absolute risk for a meaningful time period	Not applicable
Other analyses	17	Report other analyses done—eg analyses of subgroups and interactions, and sensitivity analyses	19
**Discussion**			
Key results	18	Summarise key results with reference to study objectives	19-22
Limitations	19	Discuss limitations of the study, taking into account sources of potential bias or imprecision. Discuss both direction and magnitude of any potential bias	19-24
Interpretation	20	Give a cautious overall interpretation of results considering objectives, limitations, multiplicity of analyses, results from similar studies, and other relevant evidence	19-24
Generalisability	21	Discuss the generalisability (external validity) of the study results	19-24
**Other information**			
Funding	22	Give the source of funding and the role of the funders for the present study and, if applicable, for the original study on which the present article is based	24

*Give information separately for cases and controls in case-control studies and, if applicable, for exposed and unexposed groups in cohort and cross-sectional studies.

Note: An Explanation and Elaboration article discusses each checklist item and gives methodological background and published examples of transparent reporting. The STROBE checklist is best used in conjunction with this article (freely available on the Web sites of PLoS Medicine at http://www.plosmedicine.org/, Annals of Internal Medicine at http://www.annals.org/, and Epidemiology at http://www.epidem.com/). Information on the STROBE Initiative is available at www.strobe-statement.org.

For peer review only
